# Food-grade cationic antimicrobial ε-polylysine transiently alters the gut microbial community and predicted metagenome function in CD-1 mice

**DOI:** 10.1038/s41538-017-0006-0

**Published:** 2017-12-18

**Authors:** Xiaomeng You, Jonah E. Einson, Cynthia Lyliam Lopez-Pena, Mingyue Song, Hang Xiao, David Julian McClements, David A. Sela

**Affiliations:** 1 Department of Food Science, University of Massachusetts, Amherst, MA 01003 USA; 2 Commonwealth Honors College, University of Massachusetts, Amherst, MA 01003 USA; 3 Department of Microbiology, University of Massachusetts, Amherst, MA 01003 USA; 4 Department of Microbiology & Physiological Systems and Center for Microbiome Research, University of Massachusetts Medical School, Worcester, MA 01655 USA; 5 Nestlé Nutrition, 445 State St., Fremont, MI 49413 USA

**Keywords:** Applied microbiology, Microbiota

## Abstract

Diet is an important factor influencing the composition and function of the gut microbiome, but the effect of antimicrobial agents present within foods is currently not understood. In this study, we investigated the effect of the food-grade cationic antimicrobial ε-polylysine on the gut microbiome structure and predicted metagenomic function in a mouse model. The relative abundances of predominant phyla and genera, as well as the overall community structure, were perturbed in response to the incorporation of dietary ε-polylysine. Unexpectedly, this modification to the gut microbiome was experienced transiently and resolved to the initial basal composition at the final sampling point. In addition, a differential non-random assembly was observed in the microbiomes characterized from male and female co-housed animals, although their perturbation trajectories in response to diet remain consistent. In conclusion, antimicrobial ε-polylysine incorporated into food systems transiently alters gut microbial communities in mice, as well as their predicted function. This indicates a dynamic but resilient microbiome that adapts to microbial-active dietary components.

## Food science: Food additives affect gut commensal bacteria

Despite the trend of ‘going green’, in modern societies food additives are inadvertently ingested by us on a daily basis. While these additives have all been tested for the lack of direct harm, whether they may indirectly affect our health is unclear. In the gut, a complex mixture of bacteria is finely maintained to promote beneficial immunity. Using mouse as a model, the team led by David Sela at University of Massachusetts studies shows that an anti-microbial food additive, cationic homopolymer ε-polylysine, that is meant for thwarting bacteria in packed food, can also alter the ‘balance’ of gut bacteria, and, interestingly, does so in a gender-independent manner. These findings pave the road for further studies associating food additives, gut bacteria and immune-related diseases.

## Introduction

It is estimated that the mucosal surfaces of the human gastrointestinal tract (GIT) are colonized by a total of 10^13^–10^14^ microorganisms that assemble into communities.^1^ As such, these microbial communities are diverse in form and function and often contribute to host homeostatic operations. Host–microbial interactions impact local anatomic sites along the GIT,^2–5^ as well as distal locales to participate in host energy balance and metabolism.^6–9^ In addition, there is emerging evidence that the gut microbiome participates in neurodevelopment and cognitive processes.^10–12^ Accordingly, there are several external factors that may alter the community structural trajectory and function in specific instances. These extrinsic antecedents or perturbations may be specific to host developmental stage^13–15^ and include alterations to diet or other lifestyle elements,^16–19^ microbiome-active xenobiotics, such as antibiotics,^20–22^ host genotype interactions with cultural traditions,^13^ and exposure to allochthonous microbiota and environmental chemicals.^18, 23^ Among these factors, dietary interventions are a viable strategy to maintain, restore, or enhance gut microbiota function depending on the desired outcome.^16–19^ In a particularly illustrative example of linking diet with the microbiome, European children that ingest a modern western diet (i.e. 51.8% carbohydrate, 32.8% fat, and 15.4% protein with high-saturated fat and refined carbohydrate and low fiber^24^) exhibit a significantly divergent microbial community from those living in rural Africa.^18^ Similar observations of gut communities and their response to diets rich in fat and refined carbohydrates with low fiber content has emerged as a prevailing principle of nutrition.^18, 19^

In addition to the macromolecule content, other minor components commonly incorporated in the western diet may also alter the gut microbiota.^24^ Nevertheless, it is currently poorly understood what influence this broad class of molecules may have on microbial communities and downstream host homeostatic processes. Various additives are commonly used in commercial food products to ensure safety, increase shelf life, or enhance organoleptic properties. Recently, it was reported that common food-grade emulsifiers (i.e. carboxymethylcellulose and polysorbate-80) alter the gut microbiome community, thereby potentially promoting inflammation, colitis, and metabolic syndrome.^25^ This would suggest that under certain conditions a seemingly inert food additive might influence host physiology subsequent to interacting with one’s gut microbiota. Clearly, there is a need to understand this phenomenon in greater depth.

When last enumerated in 2014 there were over 2500 additives approved for use in US food systems,^26^ with antimicrobial agents accounting for a significant fraction. Antimicrobials inhibit or retard pathogenic and spoilage microbes in foods. Although it is unknown to what extent antimicrobial activity in a food influences the resident microbes of the GIT once ingested.

One such food-grade antimicrobial is the cationic homopolymer ε-polylysine that typically consists of 25–40 l-lysine residues linked by isopeptide bonds between ε-amino and α-carboxyl groups. The US Food and Drug Administration (FDA) conferred generally regarded as safe (GRAS) status on ε-polylysine to be incorporated into various food items at levels of 10–500 ppm.^27, 28^ Accordingly, ε-polylysine exhibits broad inhibitory activity against several microorganisms, including fungi, such as *Aspergillus niger*, *Trichophyton mentagrophytes*, *Candida* spp., and *Phaggia rhodozyma*. In addition, ε-polylysine restricts a broad range of Gram-positive and Gram-negative bacterial taxa, such as *Bacillus coagulans*, *Staphylococcus aureus*, *Escherichia coli*, and *Salmonella typhimurium*.^29–31^ The antimicrobial activity of ε-polylysine has been largely attributed to its cationic charge, as this allows it to adsorb onto negatively charged microbial surfaces and disrupt the cell envelope.^32^ The cationic nature of ε-polylysine often limits its application in foods as it could interact with anionic mucins in the mouth, or spontaneously complex with negatively charged molecules to impact food integrity.^33–35^ Delivering ε-polylysine in an electrostatic complex with anionic polysaccharides, such as pectin may mitigate these limitations.^33–35^ These complexes are designed to retain ε-polylysine-mediated antimicrobial activity.

Both ε-polylysine and pectin evade digestion and absorption by the host during transit through the GIT.^36, 37^ Consequently, ε-polylysine or ε-polylysine–pectin complexes would be expected to reach the colon intact and thus available to interact with the gut microbial community. In this study, the antimicrobial food additive ε-polylysine was experimentally investigated to characterize potential perturbations to the gut microbiome. In addition, the impact of electrostatic complexation of ε-polylysine with the anionic pectin was studied as it is often delivered in this format.

## Results

### A cationic antimicrobial biopolymer influences murine gut community diversity

**Fig. 1:**
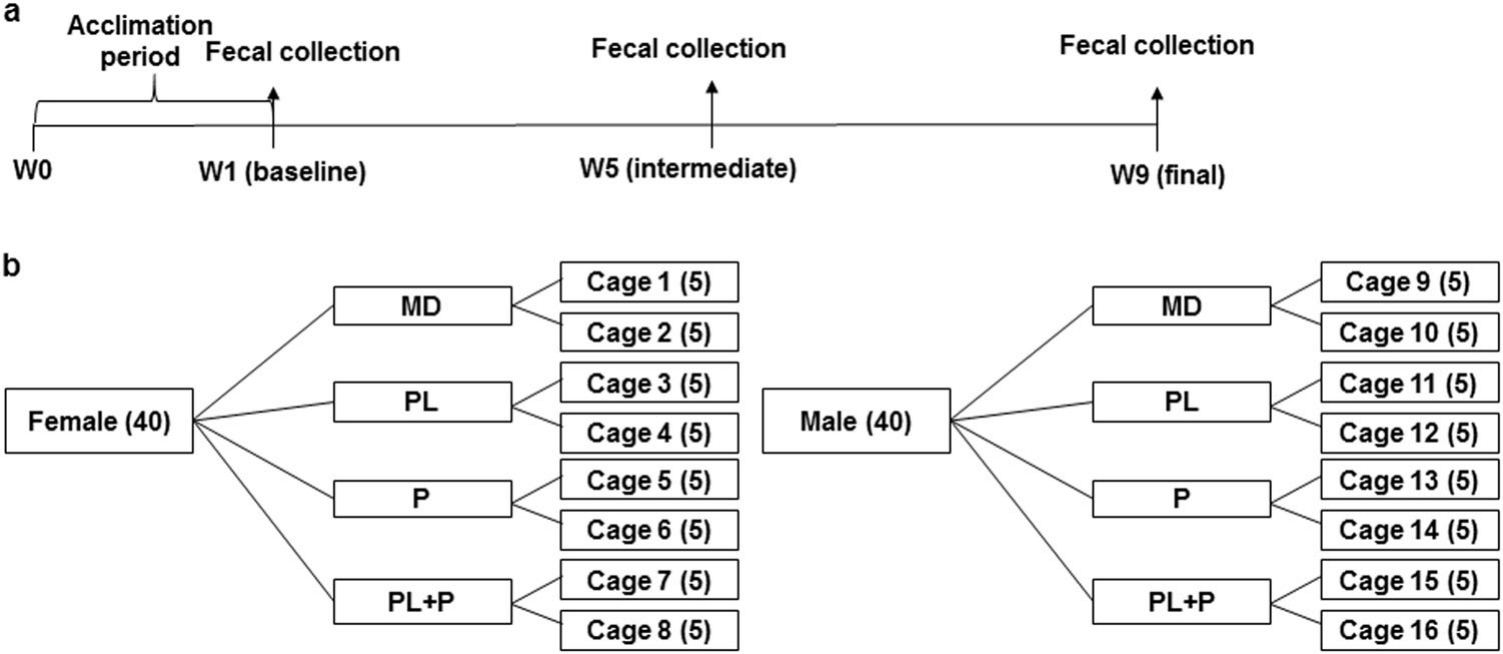
Study design of timeline (**a**) and grouping (**b**). Forty female and 40 male 6-week old CD-1 mice were randomly divided into four groups and segregated by sex and fed a 20% high-fat diet supplemented with (i) maltodextrin alone (MD), (ii) maltodextrin + ε-polylysine (PL), (iii) maltodextrin + pectin (P), and (iv) maltodextrin + ε-polylysine + pectin (PL-P). Five mice were co-housed and pooled fecal pellets from each cage were collected in 24-h metabolic cages and analyzed at three points: week 1, week 5, and week 9

40 female and 40 male 6-week old CD-1 mice were randomly divided into four groups and segregated by sex (i.e. 10 female and 10 male mice per group) and fed a 20% high-fat diet supplemented with (i) maltodextrin alone (MD) that served as the control, (ii) maltodextrin + ε-polylysine (PL), (iii) maltodextrin + pectin (P), and (iv) maltodextrin + ε-polylysine + pectin (PL-P). Previous studies showed that mice co-housed together exhibit similar gut microbial communities.^38, 39^ Thus, pooled fecal pellets from each cage were collected in 24-h metabolic cages and analyzed at three points: week 1 (baseline), week 5 (intermediate phase), and week 9 (final phase) (Fig. 1). Body weight and food consumption did not vary regardless of treatment group and across the entire experimental period.

In order to characterize phylogenetic diversity, the 16S rRNA gene V3/V4 fragment was sequenced to yield 15,739,734 quality reads following filtering.^40^ This provided a mean sample depth of 327,911 sequencing reads per bacterial community. To assess the α-diversity within a given community, the number of observed operational taxonomic units (OTUs) was calculated using weighted UniFrac.^41^ Rarefaction curves for the observed OTUs (Fig. S1) approached an asymptote independent of diet, sampling point, and sex to indicate sequencing depth sufficiently covered OTU diversity present in the communities extracted from fecal samples.

**Fig. 2:**
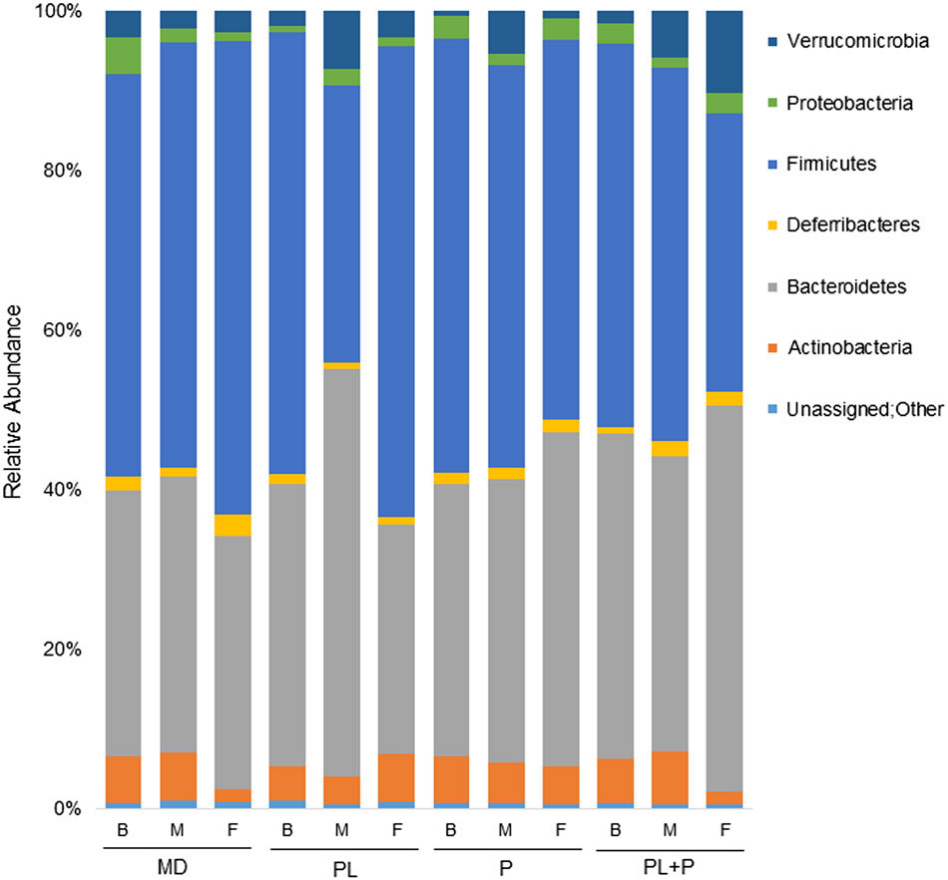
Relative abundances of bacteria phyla in response to biopolymer feeding. Pooled fecal samples were collected from two female and two male cages per group at each time point. Each bar represents the average relative abundance bacterial phyla within a treatment group during each time point with each colored box representing a bacterial phylum taxon. *B*baseline, *M* intermediate, *F* final, *MD* maltodextrin, *PL* ε-polylysine, *P* pectin, *PL+P* ε-polylysine–pectin complexes

At the phylum level, the summation of Actinobacteria, Bacteroidetes, Deferribacteres, Firmicutes, Proteobacteria, Verrucomicrobia constituted over 99% of OTUs identified in all samples analyzed. As previously reported,^42, 43^ the murine gut microbiome consists of relatively large contributions provided by the phyla Bacteroidetes and Firmicutes (Fig. 2). This is consistent with other mammalian gut communities, including humans and non-human primates.^44, 45^ However, the relative abundances of Bacteriodetes (*p* < 0.05) and Firmicutes (*p* < 0.05) were altered in response to the particular dietary biopolymer (multi-way ANOVA) (Fig. 2). Interestingly, mice fed the ε-polylysine–pectin complex exhibited an increase of Bacteriodetes at 8.82% (*p* < 0.05, multi-way ANOVA Tukey HSD post-hoc) with a corresponding decreasing of OTUs assigned to Firmicutes by 11.13% (*p* < 0.05, multi-way ANOVA Tukey HSD post-hoc). This is relative to the community structure determined in the maltodextrin-fed control group (Table S1). Moreover, the ε-polylysine (i.e. no pectin complex) fed mice exhibited a community relatively deficient for Firmicutes at the intermediate phase (i.e. 5 weeks) of the study. Remarkably, Firmicute OTUs rebounded to its initial concentration at the 9-week sampling point (baseline: 55.24%, intermediate: 34.71%, final: 59.01%, baseline vs. intermediate: *p* < 0.05, intermediate vs. final: *p* < 0.05, multi-way ANOVA Tukey HSD post-hoc) (Fig. 2 and Table S3). Exhibiting the same adaptive response, the relative fraction of Bacteriodetes OTUs transiently increased at 5 weeks of feeding and converged to initial concentrations at the final time point (baseline: 35.40%, intermediate: 51.18%, final: 28.82%, baseline vs. intermediate: *p* < 0.05, intermediate vs. final: *p* < 0.05, multi-way ANOVA Tukey HSD post-hoc) (Fig. 2 and Table S3). A similar transient surge in Verrucomicrobia occurred in mice fed pectin alone, to fall to original levels at the final sampling point (baseline: 0.59%, intermediate: 5.46%, final: 1.01%, baseline vs. intermediate: *p* < 0.05, intermediate vs. final: *p* < 0.05 multi-way ANOVA Tukey HSD post-hoc) (Fig. 2 and Table S4). In aggregate, these results indicate that specific food grade biopolymers transiently direct phyla representation within the murine gut. This was observed when both ε-polylysine and pectin were incorporated individually. However, when ε-polylysine was complexed with the anionic pectin, this phenomenon was not observed. It is noteworthy that significant population fluxes within Actinobacteria, Deferribacteres, and Proteobacteria were not observed regardless of diet (Table S5).

**Fig. 3:**
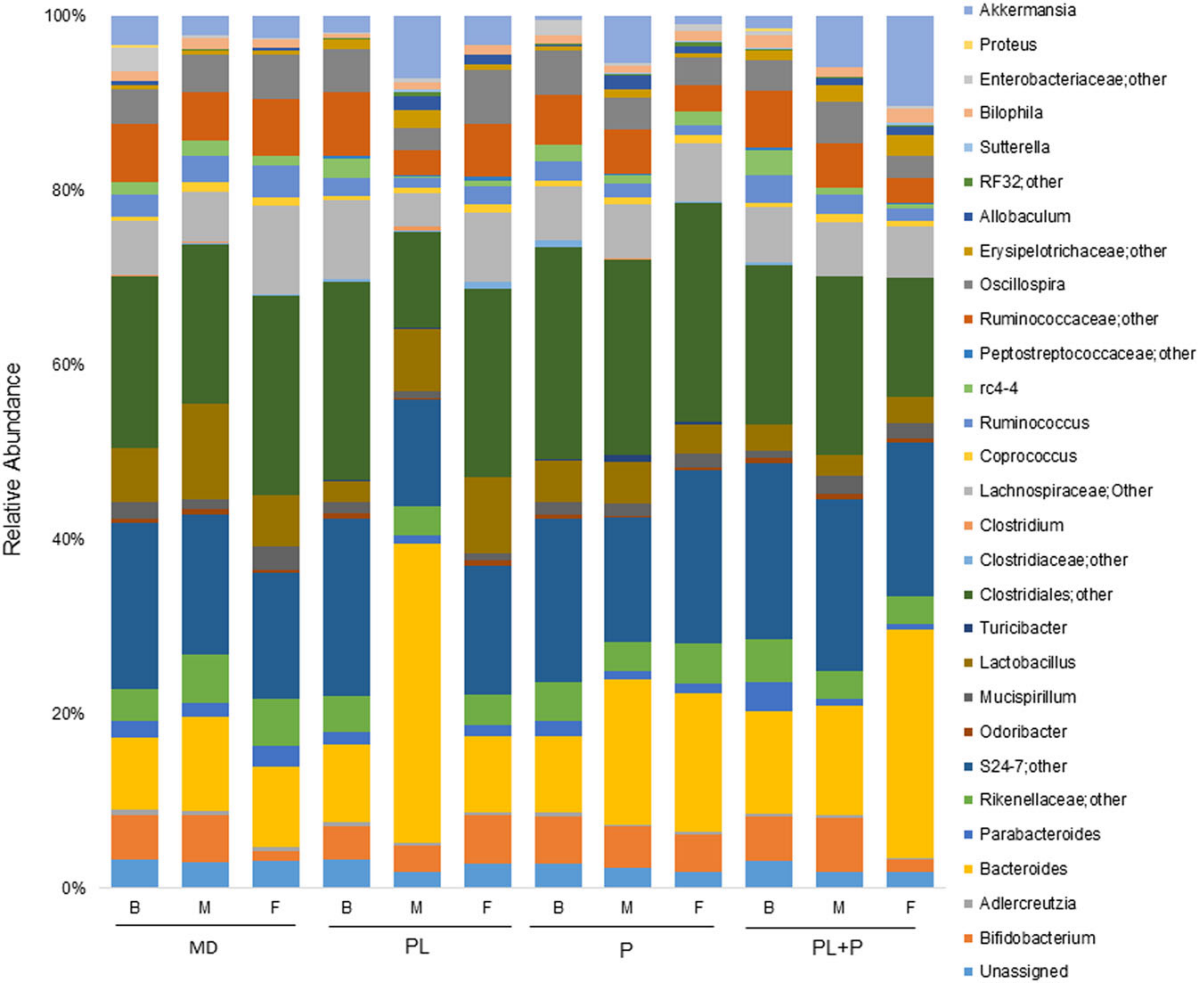
Relative abundance of bacterial genera in response to dietary biopolymers. Pooled fecal samples were collected from two female and two male cages per group at each time point. Each bar represents the average relative abundance of a treatment group during each time point and each colored box represents a bacterial genus taxon. *B* baseline, *M* intermediate, *F* final, *MD* maltodextrin, *PL* ε-polylysine, *P* pectin, *PL+P* ε-polylysine–pectin complexes

In addition to phylum-level community disruption, several bacterial genera shifted in response to dietary biopolymers. This includes members of the genus *Bacteroides* that were the most frequently encountered taxa within the mouse gut (14.32 ± 9.58% across all the samples). In total (across both sexes and sampling points), *Bacteroides* representation varied with respect to biopolymer feeding group. Mirroring the response by the phylum Bacteroidetes, ε-polylysine (*p* < 0.05, multi-way ANOVA Tukey HSD post-hoc) and ε-polylysine–pectin complexed treatment (*p* < 0.05, multi-way ANOVA Tukey HSD post-hoc) increased the *Bacteroides* spp. populations by 7.95 and 7.46%, respectively, in comparison to the maltodextrin control group (Fig. 3 and Table S6). Other bacterial populations that were modulated by feeding regimes include, *Adlercreutzia, Lactobacillus, Turicibacter*, and *Ruminococcus* (multi-way ANOVA, *p* < 0.05). Specifically, the abundance of *Adlercreutzia* decreased regardless of biopolymer relative to the maltodextrin-fed group. Their relative populations decreased 0.18%, 0.22%, 0.24% in mice fed ε-polylysine, pectin, and ε-polylysine–pectin complexes, respectively. In addition, the genus *Ruminococcus* significantly decreased by 1.39% in response to ε-polylysine and decreased by 1.44% in the pectin group. Furthermore, the ε-polylysine–pectin complexed diet significantly decreased the *Lactobacillus* content by 4.95%, reflecting overall diminishment of Firmicutes in this group. This is in contrast to the pectin diet that enriched for *Turicibacter* relative to the other three diets. A full catalog of genera differing in response to biopolymer conditions is provided in Table S6.

Maltodextrin is commonly employed as a thickening agent or filler in various nutritional applications. This polysaccharide was used in the formulation of all treatment diets, and thus served as a control to determine if maltodextrin alone would enrich for bacteria capable of hydrolyzing the α-1-4 glycosidic linkages between d-glucose residues. As such, the relative abundance of *Coprococcus* in the mouse gut was enriched while consuming maltodextrin incorporated within their food. The response trajectory included a significant increase between baseline and intermediate sampling points and between baseline and final time point (baseline: 0.56%, intermediate: 0.98%, final: 0.91%, baseline vs. intermediate: *p* < 0.05, baseline vs. final: *p* < 0.05) (Fig. 3 and Table S7).

In the ε-polylysine treatment group, the relative abundance of *Bacteroides* transiently increased, consistent with the oscillation at the phylum level (baseline: 8.85%, intermediate: 34.40%, final: 8.74%, baseline vs. intermediate: *p* < 0.05, intermediate vs. final: *p* < 0.05). A similar response was observed for *Oscillospira* (baseline: 4.96%, intermediate: 2.51%, final: 6.13%, baseline vs. intermediate: *p* < 0.05, intermediate vs. final: *p* < 0.05). This is contrasted with a transient decrease in OTUs assigned to *Ruminococcus* (baseline: 2.17%, intermediate: 0.97%, final: 2.10%, baseline vs. intermediate: *p* < 0.05, intermediate vs. final: *p* < 0.05), and *Adlercreutzia* (baseline: 0.49%, intermediate: 0.24%, final: 0.34%, baseline vs. intermediate: *p* < 0.05) (Fig. 3 and Table S8). Furthermore, *Coprococcus* spp. exhibited a sustained enrichment during feeding, with significant increases between baseline and final time point (baseline: 0.47%, intermediate: 0.71%, final: 0.91%, baseline vs. final: *p* < 0.05) (Fig. 3 and Table S8). This is consistent with the same trend observed within the maltodextrin control group. However, *Coprococcus* populations remain relatively static in mice-fed pectin and ε-polylysine–pectin complexes.

Pectin transiently enriched for the genus *Akkermansia* (baseline: 0.59%, intermediate: 5.46%, final: 1.01%, baseline vs. intermediate: *p* < 0.05, intermediate vs. final: *p* < 0.05) contributing to the observed intermediate increase of Verrucomicrobia. In contrast, *Adlercreutzia* populations were diminished in the intermediate sampling point and remained depressed in the final observation (*Adlercreutzia* baseline: 0.48%, intermediate: 0.23%, final: 0.26%, baseline vs. intermediate: *p* < 0.05). Candidate genus rc4-4 OTU representation diminished proportionally, though exhibited incomplete rebound to initial states (rc4-4 baseline: 1.94%, intermediate: 1.04%, final: 1.62%, baseline vs. intermediate: *p* < 0.05) (Fig. 3 and Table S9). Interestingly, *Ruminococcus* spp. maintained a diminishing trajectory across the feeding study with significant differences between baseline and final time points (baseline: 2.32%, intermediate: 1.63%, final: 1.14%: baseline vs. final: *p* < 0.05) (Fig. 3 and Table S9).

Although most genera did not shift in response to ε-polylysine–pectin complexes, *Ocscillospira* transiently increased prior to falling below initial levels (baseline: 3.56%, intermediate: 4.70%, final: 2.44%, intermediate vs. final: *p* < 0.05). In contrast, *Parabacteroides* populations were generally inhibited by any of the dietary treatments (baseline: 3.44%, intermediate: 0.79%, final: 0.55%, baseline vs. intermediate: *p* < 0.05, baseline vs. final: *p* < 0.05). This is similar to rc4-4 (baseline: 2.82%, intermediate: 0.77%, final: 0.48%, baseline vs. intermediate: *p* < 0.05, baseline vs. final: *p* < 0.05). In totality, gut microbiota genera respond to food grade additives at the genus level, in particular ε-polylysine (Fig. 3 and Table S10).

**Fig. 4:**
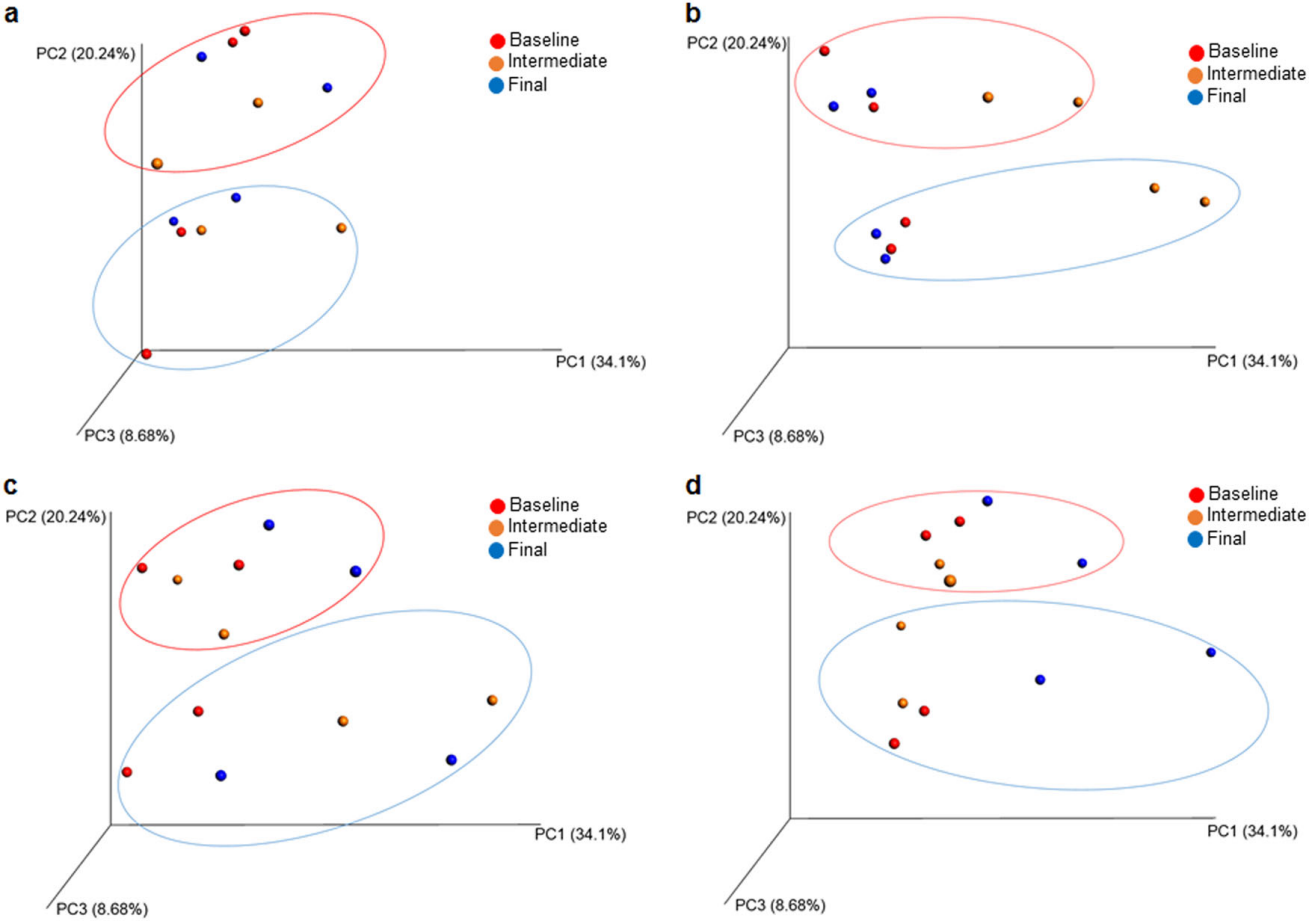
Principal coordinate analysis (PCoA) plots of microbiome response to maltodextrin (**a**), ε-polylysine (**b**), pectin (**c**) ε-polylysine-pectin complexes (**d**). PCoA plots based on weighted UniFrac distances. Each sphere represents the pooled communities from 5 mice that were co-housed during each sampling point. The red circle indicates communities extracted from female mice and blue from male mice. The red and blue boundaries are delineated arbitrarily and provided solely to aid visualization of each sex group. Principal coordinate PC1, PC2, and PC3 explain 63.02% of the total variance observed

In addition to dietary biopolymers influencing specific taxa, the structural composition of the community had discernable and non-random changes in aggregate. ANOSIM^46^ with 999 permutations was used to test significant differences between sample groups based on weighted UniFrac.^41^ As expected, maltodextrin did not significantly shift the murine gut microbial community fed this control in either female or male mice (Fig. 4a, ANOSIM with 999 permutations, *p* > 0.05). Accordingly, pectin (Fig. 4c, ANOSIM with 999 permutations, *p* > 0.05) and ε-polylysine–pectin complexes (Fig. 4d, ANOSIM with 999 permutations, *p* > 0.05) did not promote significant shifts within the community. This is in remarkable contrast to the gut microbiomes that were transiently altered by ε-polylysine alone. As observed with specific taxonomic groups at the phylum and genus levels, the community structure was perturbed at the 5-week point to be subsequently resolved at the final sampling time. This suggests that the population composition was corrected to its initial state through adaptation to the continuously fed biopolymer (Fig. 4b, ANOSIM with 999 permutations, *p* < 0.05). This was not observed in the ε-polylysine–pectin complex treated mice, indicative of a shielding interaction with the microbial community.

### ε-polylysine transiently shifts predicted metagenome function

The metagenomic potential inherent to gut microbiomes were inferred by Phylogenetic Investigation of Communities by Reconstruction of Unobserved States (PICRUSt) based on 16S rRNA phylogenetic data. A total of 6,854,103,780 observations were predicted across 6909 Kyoto Encyclopedia of Genes and Genomes (KEGG) orthology groups (KO) within the 48 gut communities that were profiled by PICRUSt. The resultant data were categorized into 254 functional pathways encompassing all of the 48 communities.

**Fig. 5:**
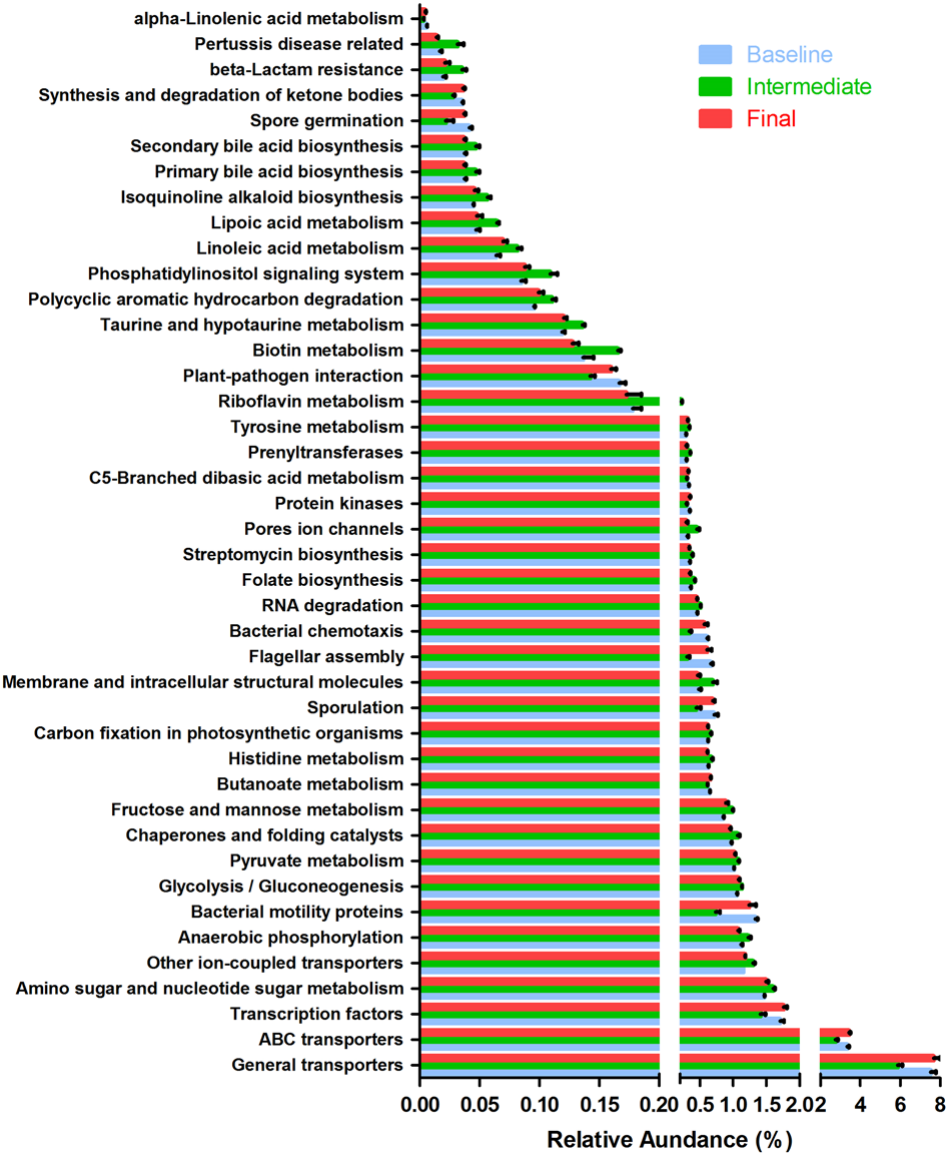
Effect of ε-polylysine on predicted metagenome function over time. The relative abundance of intermediate time points show significant difference from baseline and final time points for all pathways (*p* < 0.05)

In total, there were 44 pathways that were predicted to significantly shift transiently while mice were consuming ε-polylysine (ANOVA with Bonferroni correction, *p* < 0.05) (Fig. 5). As with alterations to taxonomic structure, the intermediate samples taken at 5 weeks displayed a significantly different profile relative to baseline and final sampling points. Among these 44 pathways or networks, 42 pathways are involved in bacterial metabolism or are otherwise predicted to mediate host–microbial interactions, with 18 pathways present at 0.5% relative abundance based on the average of the three sampling points (Fig. 5). Of these, eight pathways were predicted to decrease at 5 weeks and return to basal levels at the final sampling. Conversely, 10 predicted pathways exhibited the opposite trend and temporarily increased in abundance. Accordingly, genes and their pathways related to general solute transport (baseline: 7.53%, intermediate: 5.88%, final: 7.68%, *p* < 0.05) and ABC transporters (baseline: 3.35%, intermediate: 2.76%, final: 3.44%, *p* < 0.05) were suppressed at the intermediate community state and ultimately rebounded to baseline levels. This suggests that metabolic needs, and/or environmental concentrations of desirable solutes, may be briefly diminished prior to restoration of microbiome structure. Moreover, predicted central metabolic processes involved in carbohydrate and protein metabolisms transition to a reversible state, while the host consumes the ε-polylysine-enriched diet. This is reflected in glycolytic/fermentative pathways (baseline: 1.05%, intermediate: 1.13%, final: 1.08%, *p* < 0.05), pyruvate metabolism (baseline: 1.01%, intermediate: 1.07%, final: 1.02%, *p* < 0.05), fructose and mannose metabolisms (baseline: 0.085%, intermediate: 0.099%, final: 0.089%, *p* < 0.05), and genes associated with oxidative phosphorylation (baseline: 1.12%, intermediate: 1.23%, final: 1.08%, *p* < 0.05). The latter KO likely involved in anaerobic respiration and transiently enriched in the intermediate time point. In addition to carbohydrate catabolism, pathways associated with nitrogen flux were shifted at 5 weeks including amino sugar and nucleotide sugar metabolisms (baseline: 1.47%, intermediate: 1.60%, final: 1.50%, *p* < 0.05), and histidine metabolism (baseline: 0.062%, intermediate: 0.067%, final: 0.061%, *p* < 0.05). We had initially hypothesized that hallmarks of lysine catabolism would be enriched in the predicted metagenomes of mice-fed ε-polylysine, either in the PL or PL-P diet. However, this signal was not observed in the PICRUSt analysis, suggesting that ε-polylysine did not select bacterial populations that increased the lysine catabolic potential.

Whereas the predicted metagenomes responded to dietary ε-polylysine, no significant differences were detected in the other three feeding groups. This includes mice-fed ε-polylysine complexed with pectin, providing further support for electrostatic shielding to mitigate the anti-microbial influence of the ε-polylysine. Pectin alone does not alter the community structure or predicted function.

### Host sex influences the basal microbome but not the response trajectory

Both male and female mice were observed to determine if biopolymer activity within the microbiome is sex-dependent. Accordingly, several bacterial taxa colonized male and female animals asymmetrically and in a non-random manner. This includes the phylum Verrucomicrobia found at higher concentrations in female mice than males in aggregate (female: 4.96% of 24 samples, male: 2.63% of 24 samples, *p* < 0.05, multi-way ANOVA) (Table S11). Much of this may be accounted for by differences in *Akkermansia* populations (female: 4.50%, male: 2.63% *p* < 0.05). In addition, female mice harbored significantly greater populations of bacterial genera *Parabacteroides* (female: 2.47%, male: 0.5%, *p* < 0.05) and *Bilophila* (female: 2.13%, male: 0.01%, *p* < 0.05). In contrast, male mice were colonized by greater concentrations of *Odoribacter* (female: 0%, male: 0.92%, *p* < 0.05), *Turicibacter* (female: 0.02%, male: 0.21%, *p* < 0.05), *Clostridium* (female: 0.01%, male: 0.10%, *p* < 0.05), and candidate genus rc4-4 (female: 0.16%, male: 2.46%, *p* < 0.05) (Table S12).

**Fig. 6:**
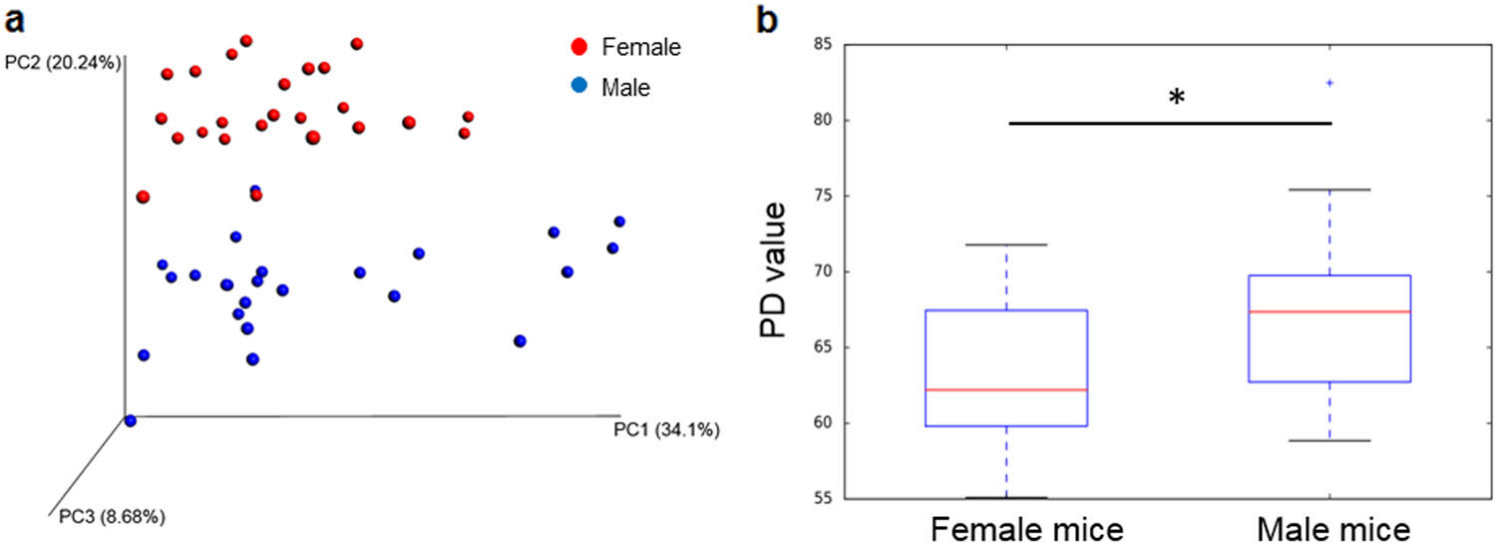
Sex difference in gut microbiome structure (**a**), and phylogenetic diversity (**b**). Red dots represents female mice microbiomes and blue dots represent those analyzed from male mice. The PCoA plot is based on weighted UniFrac distances between all OTUs identified in female and male mice. Female mice showed a significant difference from male mice by ANOSIM with 999 permutations analysis (*p* < 0.05). In **b**, microbiomes harbored in female mice exhibited a significantly lower PD value than male mice. * *p* < 0.05

In addition to taxonomic differences, there are structural differences to the community attributable to animal sex evident in UniFrac distance visualized by principal coordinate analysis (PCoA) in Fig. 6a. Accordingly, gut microbiota observed in female and male mice cluster together by sex, and in a manner more similar within their respective sex than they are to each other (Fig. 6a, ANOSIM with 999 permutations, *p* < 0.05). In addition, hierarchical clustering of microbiomes and bacterial genera based on their relative abundance exhibited a similar pattern in that bacterial communities within the same sex tend to cluster (Figs. S2 and S3). The average within group phylogenetic diversity of female male is smaller than male mice (Fig. 6b, *t*-test, *p* < 0.05), while differences were not observed between female and male mice in the total number of observed OTUs and Chao 1 index (Fig. S4).

In contrast to structural differences between female-hosted and male-hosted microbiomes, only three predicted metagenomic pathways significantly varied. This includes transcription-related operations (female: 0.0092%, male: 0.0051%, *p* < 0.05), aminoglycoside antibiotic biosynthesis (female: 0.087%, male: 0.080%, *p* < 0.05) and glycerophospholipid metabolism (female: 0.55%, male: 0.52%, *p* < 0.05). It is unclear whether these underlie expressed metabolic differences between the two host sexes. Conservation of function despite taxonomic variation is consistent with redundancy in genetic potential previously observed within other microbial communities.^46, 47^

Despite sex-dependent features, the specific effect of biopolymer treatments remains independent of sex as indicated by PCoA analysis (Fig. 3). Specifically, ε-polylysine transiently alters the murine microbiome at the intermediate sampling point regardless of sex. Whereas pectin or pectin complexed with ε-polylysine does not alter female and male mice harbored microbiota. A similar response path is evident in fluxes at the phylum level, as Bacteriodes and Firmictues representation is temporarily shifted in response to ε-polylysine in both sexes (Fig. S5). In addition, the gut microbiomes derived from both sexes exhibited the same change in Verrucomicrobia when fed pectin (Fig. S5). Furthermore, relative to the maltodextrin-fed group, the ε-polylysine–pectin complexed diet increased Bacteriodetes and diminished the Firmicutes independent of sex. These results indicate that sex-dependent traits (e.g. hormones) did not act synergistically or antagonistically with dietary biopolymers to alter community structure overall, and within major phyla.

Interestingly, dietary biopolymers may alter the genus representation that is somewhat dependent on sex. The relative abundance of *Parabacteroides* (sex*treatment *p* < 0.05, multiway ANOVA), *Clostridium* (sex*treatment *p* < 0.05, multiway ANOVA), *Coprococcus* (sex*treatment, *p* < 0.05, multiway ANOVA), and *Bilophila* (sex*treatment *p* < 0.05, multiway ANOVA) exhibited modest dependence on host sex (Fig. S6). In female mice, the *Coprococcus* content was higher in the pectin group relative to the other three groups in female mice (MD: 0.69%, PL: 0.63%, P: 0.98%, PL+P: 0.65%, MD vs. P: *p* < 0.05, PL vs. P: *p* < 0.05, P vs. PL+P: *p* < 0.05). However, in male mice, *Coprococcus* OTUs of pectin group were decreased in the microbiomes compared to the other three groups (MD: 0.94%, PL: 0.77%, P: 0.45%, PL+P: 0.78%, MD vs. P: *p* < 0.05, PL vs. P: *p* < 0.05, P vs. PL+P: *p* < 0.05). Also, *Bilophila* was reduced in female mice-fed ε-polylysine relative to ε-polylysine–pectin complexed diet (MD: 0.51%, PL+P: 1.63%, *p* < 0.05), whereas the microbiome of male mice did not exhibit this sex-linked population flux. In addition, the interactions of sex, sampling points, and biopolymer influenced the observed relative abundance of the genus *Turicibacter* (*p* < 0.05), *Clostridium* (*p* < 0.05), *Coprococcus* (*p* < 0.05), and the candidate genus *rc4-4* (*p* < 0.05).

## Discussion

It is widely recognized that dietary constituents often influence the composition and function of gut microbial communities.^16–19^ However, there is a critical scientific gap in understanding the potential interactions with additives that restrict microbial activity in foods. These substances are added to preserve food integrity during storage and are believed to be inert or benign with respect to toxicology to the consumer. In this study, we report the in vivo influence on the microbiome by a food grade antimicrobial ε-polylysine. Both ε-polylysine and pectin are not hydrolyzed or absorbed in the upper GIT,^36, 37^ thus have the opportunity to interact with resident microbial communities established along the full length of the GIT.

ε-polylysine has been deemed by the US FDA to be a GRAS antimicrobial agent to be deployed against a wide range of bacteria, yeasts, and molds.^29–31^ Thus, we hypothesized that ε-polylysine modulates specific gut microbial populations and the overall community structure during this dietary intervention. Interestingly, the microbial community adapted or compensated for ε-polylysine activity and converged on the initial equilibrium state by the final sampling. This is in contrast to the effect of broad-spectrum antibiotics on the gut microbiome. Typically an antibiotic prompts a durable shift within the microbiome that remains until removal of this pharmacological stress.^21, 48^ In contrast, ε-polylysine transiently perturbs the murine gut microbiome during the course of the dietary intervention. This is indicative of dynamic interactions between specific food structures at the host–microbial interface. One potential explanation is that the community possesses an innate resiliency towards this exogenous biopolymer rather than increasing the degradation of ε-polylysine. It is unknown if this is an irreversible adaptation or an observed oscillation between alternative stable states over the course of the dietary intervention. It would be interesting to ascertain if this is a form of acquired resistance within specific bacteria.^49^ In addition, competition between microbial populations for limited resources may contribute to this adaptation.^50^ Regardless of phylogenetic relatedness, bacteria compete for limiting nutritive requirements and potentially overlapping functional roles.^50^ In addition, there is a potential for negative feedback emanating from the host to correct disruption in host–microbial homeostasis. Thus, the host may resolve the perturbation between microbial populations to restore or preserve an unknown function.

In our results, as expected, the murine gut microbiome consists of relatively large contributions provided by the phyla Bacteroidetes and Firmicutes. Interestingly, ε-polylysine transiently increased the abundance of Bacteroidetes relative to the diminishment of Firmicutes OTUs. Bacteroidetes and Firmicutes are the primary phyla that dominate the distal gut of mammals and may be associated with local physiological outcomes,^51^ nutrient digestion and absorption,^52^ metabolic function,^44, 53^ among other operations. Thus, deviation from stable Bacteroidetes and Firmicute ratios may induce a host program that stabilizes the community structure. This putative mechanism remains to be tested with scientific rigor. A similar correction has been previously observed in obese individuals experiencing weight loss.^44^ Consistent with this hypothesis, 44 predicted metagenomic networks were transiently modulated and rebounded towards the original metabolic potential when observed at the end of the feeding trial.

In addition to antimicrobial activity, ε-polylysine has been ascribed anti-obesogenic properties by limiting pancreatic lipase activity,^54, 55^ enhancing fecal lipid excretion,^56^ and suppressing postprandial hypertriacylglyceridemia.^55^ However, in this study, differential weight gain among treatment groups was not observed in the animal or in post-sacrifice measurement of visceral fat (data not shown). It is possible that the concentration chosen for the feeding trial was insufficient to impact adiposity while consuming a high-fat diet. Moreover, the fluctuations in the microbiome within ε-polylysine-fed group might offset this anti-obesogenic effect.^44^

It is compelling that ε-polylysine complexed with pectin did not exhibit the same transient perturbation to the gut community. Often these biopolymer complexes are employed in food systems to stabilize their physicochemical properties. Interestingly, previous studies indicated that ε-polylysine–pectin complexes maintained similar antimicrobial properties as ε-polylysine in vitro.^34, 35^ This is in stark contrast to in vivo results in our mouse model as pectin clearly mitigates ε-polylysine microbial-active function. The exact mechanism by which ε-polylysine is shielded by pectin is unclear, although it is tempting to speculate that the anionic pectin shields the surface-active function of the cationic ε-polylysine.

Although pectin was not predicted to exert anti-microbial activity, some pectin preparations may enrich specific microbial population within the gut.^57, 58^ Pectin is a hetero-polysaccharide integrated into the plant cell wall and widely used in food systems as a gelling agent or a stabilizer. In the pectin treatment group, *Akkermansia* transiently increased to achieve original population levels at the end. *Akkermansia muciniphila* is considered to be beneficial in certain contexts and inversely correlates with body weight in rodents and humans studies.^59, 60^ The transient enrichment of *Akkermansia* spp. mirrors the influence of ε-polylysine on the microbiome resiliency. In order to fully determine the community response kinetics, a densely sampled temporal study is required to evaluate anti-microbial, as well as prebiotic activity.

Consistent with previous reports,^61–64^ the basal gut microbiome structure within the CD-1 mice exhibits sex-dependent differences. Interestingly, microbiota that are harbored by adult females are more similar to communities that colonize prepubescent mice of both genders than the male microbiota,^61^ indicating a potential causal relationship with androgens. In this study, we observed a distinct clustering of female and male microbiomes, further emphasizing the importance of controlling for sex differences in future studies in animal models. Despite variation in phylogenetic diversity, predicted metagenomic differences between sexes were minimal.

It is possible that cage effects influenced the composition of the microbiome. Mice were co-housed in four separate cages (two cages per sex). As mice are coprophagic it may contribute to the development of a common microbiome in cage mates.^39^ In order to minimize the potential for this confounder, we collected and analyzed pooled feces samples. Thus, compositional change in the microbial community is more likely due to dietary treatment.

## Conclusions

In this study, we investigated the influence of the food-grade antimicrobial biopolymer ε-polylysine on the compositional stability of the murine microbiota. Bacterial phylogenetic diversity within fecal extracts were ascertained by high-throughput amplicon sequencing of the 16S rRNA gene, and community function was predicted from relative abundances of taxa using PICRUSt. Our results indicate that dietary ε-polylysine transiently alters the gut microbiome prior to restoration of the initial microbial population structure at the conclusion of the feeding trial. This is indicative of a community adaptive response to ε-polylysine that our results demonstrate is active on the microbiome in vivo. This perturbation is mitigated when the cationic ε-polylysine is associated with the anionic polymer pectin to incisively link ε-polylysine physicochemical properties with function. Finally, the absolute population structure of these murine gut microbial communities was observed to be sex-dependent, but did not alter the response trajectory to the antimicrobial ε-polylysine.

## Materials and methods

### In vivo mouse feeding trial

The animal study was performed in accordance with the protocol approved by the University of Massachusetts, Amherst Institutional Animal Care and Use Committee (#2014-0079). Forty female and 40 male 6-week old CD-1 mice (36–40 g male, 29–33 g female) were obtained from Charles River Laboratories (Wilmington, MA, USA). The animals were housed in specific pathogen free cages (5 animals/cage) in an air-conditioned room (temperature 23 ± 2 °C, 50 ± 10% humidity, 12-h light–dark cycle) with ad libitum access to water and food. The mice were fed a 20% lipid diet to mimic the typical western concentrations for 1-week acclimation.^65^ Subsequent to a 1-week acclimation period, 40 female and 40 male mice were randomly divided into four treatment groups and segregated by sex (10 female and 10 male mice per group). The four dietary treatments were: (1) maltodextrin alone (MD) that served as the control, (2) maltodextrin + ε-polylysine (PL), (3) maltodextrin + pectin (P), and (4) maltodextrin + ε-polylysine + pectin (PL-P). Maltodextrin with a dextrose equivalent of ~18 (Maltrin^®^ M180) was provided by the Grain Processing Corporation (Muscatine, IA, USA). ε-Polylysine was purchased from Wilshire Technologies, Inc. (Princeton, NJ, USA). High-methoxyl pectin was donated by TIC Gums (White Marsh, MD, USA). The biopolymer solutions were converted into powders and incorporated into the 20% lipid mouse diet as described previously.^27, 65^ The amount of ε-polylysine incorporated was based on estimating a likely adult exposure level to ε-polylysine in beverages. The maximum concentration of ε-polylysine as a preservative is 0.025% w/w.^66^ The daily dosage (g/g body weight) of ε-polylysine that were fed to the mice was based on the average American body weight^67^ and on the average *per capita* annual consumption of soft drinks in the United States,^68^ which is estimated to be 1.4*10^−6^ g/g body weight. Previous studies have demonstrated that a mass ratio of 20:1 pectin-to-polylysine leads to electrostatic complexes that retain their antimicrobial efficacy while inhibiting precipitation.^33–35^ Therefore, the final concentration of biopolymers exposed to mice are: (1) MD, 1.4*10^−5^ g/g body weight, (2) MD, 1.4*10^−5^ g/g body weight, PL, 1.4*10^−6^ g/g body weight, (3) MD, 1.4*10^−5^ g/g body weight, P, 2.8*10^−5^ g/g body weight, and (4) MD, 1.4*10^−5^ g/g body weight, PL, 1.4*10^−6^ g/g body weight, P, 2.8*10^−5^ g/g body weight. The amount of powders incorporated into the mice diet were made weekly based on calculation of the exposure level, the moisture of the powder from the spray dryer, and the average mice body weight of previous week. Pooled fecal pellets from each cage were freshly collected in 24-h metabolic cages and analyzed by 16s rRNA gene sequencing for gut microbiome at three time points: week 1 (baseline), week 5 (middle phase), and week 9 (final phase) subsequent to collection and storage at −80 °C (Fig. 1).

### Phylogenetic profiling by sequencing of the 16S rRNA gene amplicon

Total DNA was extracted from fecal pellets with the QIAamp DNA Stool Mini Kit (Qiagen, Valencia, CA, USA) following the manufacturer’s protocol with the addition of a bead-beating step (FastPrep-24^TM^ 5G MP Biomedicals Inc., USA). The concentration and quality of the recovered DNA was estimated with a NanoDrop Spectrophotometer (Thermo Scientific, Waltham, MA, USA). PCR was performed to amplify the 16S rRNA gene marker using primers that bound the V3 and V4 regions, which also incorporates the Illumina overhang adaptor. The primer set was developed by Illumina (FwOvAd_341F 5’TCGTCGGCAGCGTCAGATGTGTA TAAGAGACAGCCTACGGGNGGCWGCAG) and

(ReOvAd_785R 5’GTCTCGTGGGCTCGGAGATGTGTATAAGAGACAGGACTA CHVGGGTATCTAATCC).^69^ PCRs were performed in a 96-well format on a Veriti thermal cycler (Life technology, Carlsbad, CA, USA) using 2x KAPA HiFi Hotstart ReadyMix (KAPA Biosystem, Wilmington, MA, USA). AMPure XP beads (Beckman Coulter, Danvers, MA, USA) were used to purify the V3/V4 fragment amplicon from free primers and other contaminants. A second PCR attached dual indices and Illumina sequencing adapters using the Nextera XT Index Kit (Illumina, San Diego, CA, USA) with an additional round of AMPure XP bead purification. PCR products were quantified with the Qubit dsDNA BR Assay (Life technology, Carlsbad, CA, USA) and amplicon quality verified by DNA analysis ScreenTape Assay on Tape Station 2200 (Agilent Technologies, Santa Clara, CA, USA). PCR products were pooled in equimolar concentration and diluted to 4 nM and denatured immediately prior to sequencing on an Illumina MiSeq (pair-end; V3; 5% PhiX) (Illumina, San Diego, CA, USA).

### Informatic and statistical analyses

Raw Illumina fastq files were quality filtered and analyzed using the quantitative insights into microbial ecology (QIIME) software pipeline v1.9.1.^70^ Reads were truncated at any site containing more than three consecutive bases receiving a quality score <1e−5, and discarding reads containing one or more ambiguous base calls, as were truncated reads of <190 nt. OTUs were assigned in QIIME using UCLUST^71^ with a threshold of 97% pairwise identity. Open reference OTU picking was performed using a subset of the greengenes bacterial 16S rRNA database (13_8 release),^40^ filtered to remove incomplete and unannotated taxonomies. Bacterial OTUs were classified taxonomically using a QIIME-based wrapper of UCULUST, against the Greengenes 16S rRNA database using a 0.50 confidence threshold for taxonomic assignment. Bacterial 16S rRNA gene sequences were aligned using PyNAST^72^ against the greengenes core set filtered at 97% similarity with chimera sequences identified and removed using ChimeraSlayer^73^ with the resultant alignment yielding a phylogenetic tree using FastTree.^74^ OTUs representing less than 0.01% of the filtered read pool was removed to avoid inflated estimates of diversity,^75^ as were quality-filtered samples containing less than 10 sequences.

Alpha-diversity (within-sample species richness) and beta-diversity (between-sample community dissimilarity) estimates were calculated using weighted UniFrac^41^ distance between samples for bacterial 16S rRNA reads (evenly sampled at 300 reads per sample). Principal coordinates were computed from the resulting distance matrices to be visualized as 3D PCoA plots. Hierarchical clustering analyses were based on relative abundance of OTUs and heat map graphics were generated in R with heatmap.2 library package. To determine whether metadata group contained differences in phylogenetic or species diversity, ANOSIM^46^ with 999 permutations was used to test significant differences between sample groups based on weighted UniFrac^41^ distance matrices. Mice were grouped according to the categorical independent variables described in the metadata and include: treatment (MD, PL, P, PL-P), time point (baseline, intermediate, final), and sex. To see the effect of treatment, time point, sex on relative abundance of taxa, multi-way ANOVA (treatment, time point, sex) was performed with Tukey’s post hoc test. Results are presented as mean ± SD.

Metagenomic functional shifts in communities were predicted using PICRUSt. OTUs were first normalized by dividing each OTU by the predicted 16S copy number abundance and then aligned to the greengenes 16S rRNA database using a closed reference picking protocol within PICRUSt.^76^ Statistical tests were used to compare functional groups within the STAMP software environment, and Bonferroni correction was performed for multiple analyses.^77^ For all analyses, statistical significance was declared if the *p*-value < 0.05.

### Availability of supporting data

The sequence data set supporting the results of this article is available in Qiita microbial study management platform under study ID 11118.

## Electronic supplementary material

Supplemental tables

Supplemental figures

## Electronic supplementary material

**Supplementary information** accompanies the paper on the *npj Science of Food* website (10.1038/s41538-017-0006-0).

## References

[1] Gill SR (2006). Metagenomic analysis of the human distal gut microbiome. Science.

[2] Kennedy PJ, Cryan JF, Dinan TG, Clarke G (2014). Irritable bowel syndrome: a microbiome-gut-brain axis disorder?. World J. Gastroenterol..

[3] Round JL, Mazmanian SK (2009). The gut microbiota shapes intestinal immune responses during health and disease. Nat. Rev. Immunol..

[4] Gareau MG, Sherman PM, Walker WA (2010). Probiotics and the gut microbiota in intestinal health and disease. Nat. Rev. Gastroenterol. Hepatol..

[5] Maslowski KM (2009). Regulation of inflammatory responses by gut microbiota and chemoattractant receptor GPR43. Nature.

[6] Ussar S (2015). Interactions between gut microbiota, host genetics and diet modulate the predisposition to obesity and metabolic syndrome. Cell Metab..

[7] Cani PD (2008). Changes in gut microbiota control metabolic endotoxemia-induced inflammation in high-fat diet-induced obesity and diabetes in mice. Diabetes.

[8] Vijay-Kumar M (2010). Metabolic syndrome and altered gut microbiota in mice lacking Toll-like receptor 5. Science.

[9] Cani PD, Delzenne NM (2009). The role of the gut microbiota in energy metabolism and metabolic disease. Curr. Pharm. Des..

[10] O’Mahony SM, Clarke G, Borre YE, Dinan TG, Cryan JF (2015). Serotonin, tryptophan metabolism and the brain-gut-microbiome axis. Behav. Brain Res..

[11] Diaz Heijtz R (2011). Normal gut microbiota modulates brain development and behavior. Proc. Natl. Acad. Sci. USA.

[12] Cryan JF, Dinan TG (2012). Mind-altering microorganisms: the impact of the gut microbiota on brain and behaviour. Nat. Rev. Neurosci..

[13] Yatsunenko T (2012). Human gut microbiome viewed across age and geography. Nature.

[14] Walker WA (2013). Initial intestinal colonization in the human infant and immune homeostasis. Ann. Nutr. Metab..

[15] Palmer C, Bik EM, DiGiulio DB, Relman DA, Brown PO (2007). Development of the human infant intestinal microbiota. PLoS Biol..

[16] David LA (2014). Diet rapidly and reproducibly alters the human gut microbiome. Nature.

[17] Claesson MJ (2012). Gut microbiota composition correlates with diet and health in the elderly. Nature.

[18] De Filippo C (2010). Impact of diet in shaping gut microbiota revealed by a comparative study in children from Europe and rural Africa. Proc. Natl. Acad. Sci. USA.

[19] Wu GD (2011). Linking long-term dietary patterns with gut microbial enterotypes. Science.

[20] Cho I (2012). Antibiotics in early life alter the murine colonic microbiome and adiposity. Nature.

[21] Dethlefsen L, Relman DA (2011). Incomplete recovery and individualized responses of the human distal gut microbiota to repeated antibiotic perturbation. Proc. Natl. Acad. Sci. USA.

[22] Dethlefsen L, Huse S, Sogin ML, Relman DA (2008). The pervasive effects of an antibiotic on the human gut microbiota, as revealed by deep 16S rRNA sequencing. PLoS Biol..

[23] Lang JM, Eisen JA, Zivkovic AM (2014). The microbes we eat: abundance and taxonomy of microbes consumed in a day’s worth of meals for three diet types. PeerJ.

[24] Cordain L (2005). Origins and evolution of the Western diet: health implications for the 21st century. Am. J. Clin. Nutr..

[25] Chassaing B (2015). Dietary emulsifiers impact the mouse gut microbiota promoting colitis and metabolic syndrome. Nature.

[26] Carocho M, Barreiro MF, Morales P, Ferreira ICFR (2014). Adding molecules to food, pros and cons: a review on synthetic and natural food additives. Compr. Rev. Food Sci. Food Saf..

[27] Lopez-Pena CL, Song M, Xiao H, Decker EA, McClements DJ (2015). Potential impact of biopolymers (ε-polylysine and/or pectin) on gastrointestinal fate of foods: In vitro study. Food Res. Int..

[28] Chheda AH, Vernekar MR (2015). A natural preservative ε-poly-L-lysine: fermentative production and applications in food industry. Int. Food Res. J..

[29] Lopez-Pena CL (2016). Impact of epsilon-polylysine and pectin on the potential gastrointestinal fate of emulsified lipids: In vitro mouth, stomach and small intestine model. Food Chem..

[30] Yoshida T, Nagasawa T (2003). Epsilon-poly-L-lysine: microbial production, biodegradation and application potential. Appl. Microbiol. Biotechnol..

[31] Geornaras I, Yoon Y, Belk KE, Smith GC, Sofos JN (2007). Antimicrobial activity of epsilon-polylysine against Escherichia coli O157:H7, Salmonella Typhimurium, and Listeria monocytogenes in various food extracts. J. Food Sci..

[32] Hyldgaard M (2014). The antimicrobial mechanism of action of epsilon-poly-l-lysine. Appl. Environ. Microbiol..

[33] Lopez-Pena CL, McClements DJ (2014). Optimizing delivery systems for cationic biopolymers: competitive interactions of cationic polylysine with anionic kappa-carrageenan and pectin. Food Chem..

[34] Chang Y, McLandsborough L, McClements DJ (2012). Cationic antimicrobial (epsilon-polylysine)-anionic polysaccharide (pectin) interactions: influence of polymer charge on physical stability and antimicrobial efficacy. J. Agric. Food Chem..

[35] Chang Y, McLandsborough L, McClements DJ (2011). Physicochemical properties and antimicrobial efficacy of electrostatic complexes based on cationic epsilon-polylysine and anionic pectin. J. Agric. Food Chem..

[36] Holloway WD, Tasman-Jones C, Maher K (1983). Pectin digestion in humans. Am. J. Clin. Nutr..

[37] Hiraki J (2003). Use of ADME studies to confirm the safety of ε-polylysine as a preservative in food. Regul. Toxicol. Pharmacol..

[38] Campbell JH (2012). Host genetic and environmental effects on mouse intestinal microbiota. ISME J..

[39] Lees H (2014). Age and microenvironment outweigh genetic influence on the Zucker rat microbiome. PLoS One.

[40] DeSantis TZ (2006). Greengenes, a chimera-checked 16S rRNA gene database and workbench compatible with ARB. Appl. Environ. Microbiol..

[41] Lozupone C, Knight R (2005). UniFrac: a new phylogenetic method for comparing microbial communities. Appl. Environ. Microbiol..

[42] Ley RE (2005). Obesity alters gut microbial ecology. Proc. Natl. Acad. Sci. USA.

[43] Turnbaugh PJ, Backhed F, Fulton L, Gordon JI (2008). Diet-induced obesity is linked to marked but reversible alterations in the mouse distal gut microbiome. Cell Host Microbe.

[44] Ley RE, Turnbaugh PJ, Klein S, Gordon JI (2006). Microbial ecology: human gut microbes associated with obesity. Nature.

[45] Ochman H (2010). Evolutionary relationships of wild hominids recapitulated by gut microbial communities. PLoS Biol..

[46] Clarke KR (1993). Non-parametric multivariate analyses of changes in community structure. Austral. J. Ecol..

[47] Turnbaugh PJ (2007). The human microbiome project. Nature.

[48] Jakobsson HE (2010). Short-term antibiotic treatment has differing long-term impacts on the human throat and gut microbiome. PLoS One.

[49] Gunn JS, Miller SI (1996). PhoP-PhoQ activates transcription of pmrAB, encoding a two-component regulatory system involved in Salmonella typhimurium antimicrobial peptide resistance. J. Bacteriol..

[50] Lozupone CA, Stombaugh JI, Gordon JI, Jansson JK, Knight R (2012). Diversity, stability and resilience of the human gut microbiota. Nature.

[51] Singh P (2015). Intestinal microbial communities associated with acute enteric infections and disease recovery. Microbiome.

[52] Duncan SH (2008). Human colonic microbiota associated with diet, obesity and weight loss. Int. J. Obes..

[53] Cani PD, Delzenne NM (2009). Interplay between obesity and associated metabolic disorders: new insights into the gut microbiota. Curr. Opin. Pharmacol..

[54] Tsujita T, Takaichi H, Takaku T, Aoyama S, Hiraki J (2006). Antiobesity action of epsilon-polylysine, a potent inhibitor of pancreatic lipase. J. Lipid Res..

[55] Kido Y (2003). Epsilon-polylysine inhibits pancreatic lipase activity and suppresses postprandial hypertriacylglyceridemia in rats. J. Nutr..

[56] Hosomi R (2015). Dietary varepsilon-polylysine decreased serum and liver lipid contents by enhancing fecal lipid excretion irrespective of increased hepatic fatty acid biosynthesis-related enzymes activities in rats. Prev. Nutr. Food Sci..

[57] Martens EC (2011). Recognition and degradation of plant cell wall polysaccharides by two human gut symbionts. PLoS Biol..

[58] Koropatkin NM, Cameron EA, Martens EC (2012). How glycan metabolism shapes the human gut microbiota. Nat. Rev. Microbiol..

[59] Santacruz A (2010). Gut microbiota composition is associated with body weight, weight gain and biochemical parameters in pregnant women. Br. J. Nutr..

[60] Everard A (2011). Responses of gut microbiota and glucose and lipid metabolism to prebiotics in genetic obese and diet-induced leptin-resistant mice. Diabetes.

[61] Yurkovetskiy L (2013). Gender bias in autoimmunity is influenced by microbiota. Immunity.

[62] Bolnick DI (2014). Individual diet has sex-dependent effects on vertebrate gut microbiota. Nat. Commun..

[63] Markle JG (2013). Sex differences in the gut microbiome drive hormone-dependent regulation of autoimmunity. Science.

[64] Clarke G (2013). The microbiome-gut-brain axis during early life regulates the hippocampal serotonergic system in a sex-dependent manner. Mol. Psychiatry.

[65] Xiao H (2008). Green tea polyphenols inhibit colorectal aberrant crypt foci (ACF) formation and prevent oncogenic changes in dysplastic ACF in azoxymethane-treated F344 rats. Carcinogenesis.

[66] FDA. *Agency Response Letter GRAS Notice No. GRN 000336* (U.S. Food and Drug Administration). Retrieved Oct 9, 2017. https://www.fda.gov/Food/IngredientsPackagingLabeling/GRAS/NoticeInventory/ucm271325.htm (2011).

[67] CDC. *Anthropometric Reference Data for Children and Adults: United States, 2007–2010* (Centers for Disease Control and Prevention). Retrieved Oct 9, 2017. https://www.cdc.gov/nchs/data/series/sr_11/sr11_252.pdf (2010).

[68] Mintel. *Total US Retail Sales of Packages Carbonated Soft Drinks, By Segment, At Current Prices (2011 and 2013)* (Mintel Group Ltd, US Carbonated Soft Drinks market report, 2014).

[69] Yasir M (2015). Comparison of the gut microbiota of people in France and Saudi Arabia. Nutr. Diabetes.

[70] Caporaso JG (2010). QIIME allows analysis of high-throughput community sequencing data. Nat. Methods.

[71] Edgar RC (2010). Search and clustering orders of magnitude faster than BLAST. Bioinformatics.

[72] Caporaso JG (2010). PyNAST: a flexible tool for aligning sequences to a template alignment. Bioinformatics.

[73] Haas BJ (2011). Chimeric 16S rRNA sequence formation and detection in Sanger and 454-pyrosequenced PCR amplicons. Genome Res..

[74] Price MN, Dehal PS, Arkin AP (2010). FastTree 2—approximately maximum-likelihood trees for large alignments. PLoS One.

[75] Bokulich NA (2013). Quality-filtering vastly improves diversity estimates from Illumina amplicon sequencing. Nat. Methods.

[76] Langille MG (2013). Predictive functional profiling of microbial communities using 16S rRNA marker gene sequences. Nat. Biotechnol..

[77] Parks DH, Tyson GW, Hugenholtz P, Beiko RG (2014). STAMP: statistical analysis of taxonomic and functional profiles. Bioinformatics.

