# Supplementary material for: Food-grade cationic antimicrobial ε-polylysine transiently alters the gut microbial community and predicted metagenome function in CD-1 mice
Source: NPJ Sci Food. 2017 Dec 18;1:8. doi: 10.1038/s41538-017-0006-0 (PMC6550245; doi:10.1038/s41538-017-0006-0)
Supplement: Supplementary file 1 — Supplemental tables [file 41538_2017_6_MOESM1_ESM.pdf]

Table S1 Relative abundance of bacteria phylum between different biopolymer treatments in CD-1 mice

	Tx1 MD		Tx2 PL		Tx3 P		Tx4 PL+P		
	mean	SD	mean	SD	mean	SD	mean	SD	P value
Unassigned;Other	0.86%	0.44%	0.78%	0.42%	0.69%	0.35%	0.59%	0.29%	Tx1 vs Tx4: p=0.001
Actinobacteria	4.45%	6.08%	4.60%	2.21%	5.21%	3.20%	4.59%	3.65%	p=0.972
Bacteroidetes	33.23%	7.80%	38.47%	10.09%	37.12%	9.86%	42.05%	7.31%	Tx1 vs Tx4: p=0.008
Deferribacteres	1.89%	1.24%	1.00%	0.93%	1.49%	0.96%	1.51%	1.91%	p=0.474
Firmicutes	54.34%	9.15%	49.65%	11.64%	50.87%	8.08%	43.21%	9.34%	Tx1 vs Tx4: p=0.006
Proteobacteria	2.50%	3.61%	1.32%	0.97%	2.26%	1.95%	2.13%	1.59%	p=0.618
Verrucomicrobia	2.72%	3.03%	4.18%	4.21%	2.35%	2.99%	5.92%	5.64%	p=0.099

Table S2 Relative abundance of bacteria phylum between different time points in CD-1 mice fed with MD

	Baseline		Intermediate		Final		
	mean	SD	mean	SD	mean	SD	P value
Unassigned;Other	0.75%	0.26%	1.05%	0.71%	0.80%	0.23%	B vs M: p=0.005 M vs F: p=0.020
Actinobacteria	5.79%	8.02%	5.99%	7.32%	1.58%	1.00%	p=0.578
Bacteroidetes	33.30%	11.08%	34.63%	6.81%	31.76%	6.98%	p=0.804
Deferribacteres	1.87%	1.57%	1.03%	0.96%	2.76%	0.55%	p=0.234
Firmicutes	50.39%	9.10%	53.30%	8.14%	59.33%	10.13%	p=0.318
Proteobacteria	4.57%	5.92%	1.79%	1.62%	1.16%	1.12%	p=0.454
Verrucomicrobia	3.34%	4.69%	2.21%	1.91%	2.61%	2.65%	p=0.808

Table S3 Relative abundance of bacteria genera between different time points in CD-1 mice fed with PL+MD

	Baseline mean	SD	Intermediate mean	SD	Final mean	SD	P value
Unassigned;Other	0.95%	0.36%	0.57%	0.28%	0.81%	0.59%	p=0.051
Actinobacteria	4.37%	2.09%	3.41%	1.76%	6.03%	2.42%	p=0.230
Bacteroidetes	35.40%	2.49%	51.18%	3.77%	28.82%	0.90%	B vs M: p=0.000 B vs F: p=0.029 M vs F: p=0.000
Deferribacteres	1.31%	1.18%	0.83%	0.52%	0.85%	1.13%	p=0.634
Firmicutes	55.24%	3.80%	34.71%	3.68%	59.01%	3.50%	B vs M: p=0.000 M vs F: p=0.000
Proteobacteria	0.77%	0.91%	2.08%	0.45%	1.11%	1.09%	p=0.73
Verrucomicrobia	1.96%	2.02%	7.23%	5.22%	3.37%	3.73%	p=0.276

Table S4 Relative abundance of bacteria genera between different time points in CD-1 mice fed with P+MD

	Baseline mean	SD	Intermediate mean	SD	Final mean	SD	P value
Unassigned;Other	0.70%	0.37%	0.78%	0.40%	0.60%	0.34%	p=0.489
Actinobacteria	5.93%	3.44%	5.04%	3.22%	4.66%	3.77%	p=0.873
Bacteroidetes	34.05%	6.05%	35.45%	13.82%	41.86%	9.08%	p=0.455
Deferribacteres	1.44%	1.31%	1.40%	0.47%	1.64%	1.17%	p=0.934
Firmicutes	54.44%	4.57%	50.53%	11.09%	47.64%	8.02%	p=0.584
Proteobacteria	2.84%	2.67%	1.34%	0.81%	2.59%	2.10%	p=0.598
Verrucomicrobia	0.59%	0.82%	5.46%	3.49%	1.01%	0.74%	B vs M: p=0.002 M vs F: p=0.004

Table S5 Relative abundance of bacteria genera between different time points in CD-1 mice fed with PL+P+MD

	Baseline mean	SD	Intermediate mean	SD	Final mean	SD	P value
Unassigned;Other	0.78%	0.28%	0.51%	0.17%	0.49%	0.37%	p=0.348
Actinobacteria	5.42%	2.45%	6.63%	4.94%	1.70%	0.93%	p=0.229
Bacteroidetes	40.89%	3.37%	36.97%	6.80%	48.29%	7.09%	p=0.053
Deferribacteres	0.67%	0.73%	1.99%	1.43%	1.86%	3.06%	p=0.629
Firmicutes	48.11%	4.99%	46.69%	6.06%	34.83%	10.78%	p=0.120
Proteobacteria	2.62%	1.61%	1.32%	1.26%	2.45%	1.96%	p=0.283
Verrucomicrobia	1.50%	1.90%	5.88%	5.61%	10.38%	5.41%	p=0.140

Table S6 Relative abundance of bacteria genera between different biopolymer treatments in CD-1 mice

	Tx1<br>MD<br>mean	SD	Tx2<br>PL<br>mean	SD	Tx3<br>P<br>mean	SD	Tx4<br>PL+P<br>mean	SD	P value
Unassigned	3.09%	0.85%	2.64%	0.87%	2.33%	0.71%	2.24%	0.97%	Tx vsTx3: p=0.021<br>Tx1vsTx4: p=0.009
Bifidobacterium	3.86%	5.84%	4.13%	2.11%	4.81%	3.10%	4.19%	3.57%	p=0.950
Adlercreutzia	0.54%	0.26%	0.36%	0.18%	0.32%	0.16%	0.30%	0.12%	Tx1vsTx2: p=0.049<br>Tx1vsTx3: p=0.015<br>Tx1vsTx4: p=0.006
Bacteroides	9.38%	3.88%	17.33%	13.03%	13.75%	8.58%	16.84%	9.51%	Tx1vsTx2: p=0.018<br>Tx1vsTx4: p=0.028
Parabacteroides	1.98%	2.05%	1.16%	0.85%	1.26%	1.05%	1.60%	2.11%	p=0.155
Rikenellaceae;other	4.91%	2.39%	3.65%	1.54%	4.10%	1.55%	3.80%	1.39%	p=0.239
S24-7;other	16.47%	4.53%	15.82%	4.42%	17.63%	3.73%	19.14%	4.82%	p=0.209
Odoribacter	0.49%	0.63%	0.43%	0.68%	0.33%	0.41%	0.60%	0.74%	p=0.414
Mucispirillum	1.89%	1.24%	1.00%	0.93%	1.49%	0.96%	1.51%	1.91%	p=0.474
Lactobacillus	7.73%	4.86%	6.15%	4.33%	4.28%	2.65%	2.78%	2.16%	Tx1vsTx4: p=0.013
Turicibacter	0.01%	0.04%	0.04%	0.12%	0.42%	0.68%	0.00%	0.00%	Tx1vsTx3: p=0.002<br>Tx2vsTx3: p=0.004<br>Tx3vsTx4: p=0.001
Clostridiales;other	20.26%	4.89%	18.39%	5.81%	23.94%	5.06%	17.51%	4.17%	Tx2vsTx3: p=0.028<br>Tx3vsTx4: p=0.009
Clostridiaceae;other	0.15%	0.08%	0.43%	0.69%	0.29%	0.69%	0.11%	0.10%	p=0.442
Clostridium	0.01%	0.01%	0.16%	0.37%	0.04%	0.11%	0.01%	0.01%	p=0.044
Lachnospiraceae;Other	7.38%	2.63%	6.95%	2.83%	6.41%	1.74%	6.09%	1.37%	p=0.155
Coprococcus	0.82%	0.30%	0.70%	0.26%	0.71%	0.32%	0.71%	0.32%	p=0.317
Ruminococcus	3.14%	0.97%	1.75%	0.61%	1.70%	0.71%	2.26%	1.76%	Tx1vsTx2: p=0.020<br>Tx1vsTx3: p=0.015
rc4-4	1.40%	1.64%	0.95%	1.42%	1.53%	1.53%	1.36%	1.97%	P=0.284
Peptostreptococcaceae;other	0.01%	0.02%	0.34%	0.49%	0.03%	0.05%	0.17%	0.41%	p=0.093

Table S6 Relative abundance of bacteria genera between different biopolymer treatments in CD-1 mice (continued)

	Tx1		Tx2		Tx3		Tx4		
	MD		PL		P		PL+P		
	mean	SD	mean	SD	mean	SD	mean	SD	P value
Ruminococcaceae;other	6.26%	1.47%	5.44%	2.43%	4.60%	2.18%	4.85%	2.28%	Tx1vsTx2: p=0.016<br>Tx1vsTx3: p=0.011
Oscillospira	4.41%	0.94%	4.53%	1.76%	3.97%	1.69%	3.57%	1.34%	p=0.144
RF32;other	0.05%	0.08%	0.22%	0.37%	0.23%	0.46%	0.16%	0.18%	p=0.226
Sutterella	0.00%	0.00%	0.10%	0.31%	0.11%	0.23%	0.13%	0.21%	p=0.510
Bilophila	1.14%	1.28%	0.77%	0.94%	0.98%	1.06%	1.37%	1.51%	Tx2vsTx4:p=0.031
Enterobacteriaceae;other	1.08%	3.05%	0.21%	0.24%	0.89%	1.87%	0.32%	0.51%	p=0.602
Proteus	0.11%	0.35%	0.00%	0.01%	0.00%	0.00%	0.09%	0.30%	p=0.566
Akkermansia	2.72%	3.03%	4.18%	4.21%	2.35%	2.99%	5.92%	5.64%	p=0.099

Table S7 Relative abundance of bacteria genera between different time points in CD-1 mice fed with MD

	Baseline mean	SD	Intermediate mean	SD	Final mean	SD	P value
Unassigned	3.26%	1.32%	2.87%	0.54%	3.14%	0.71%	p=0.740
Bifidobacterium	5.10%	7.52%	5.43%	7.20%	1.06%	0.90%	p=0.569
Adlercreutzia	0.63%	0.45%	0.52%	0.15%	0.48%	0.09%	p=0.725
Bacteroides	8.17%	2.16%	10.79%	6.19%	9.18%	2.74%	p=0.720
Parabacteroides	1.92%	2.14%	1.55%	1.49%	2.46%	2.83%	p=0.673
Rikenellaceae;other	3.63%	2.03%	5.67%	1.96%	5.44%	3.11%	p=0.447
S24-7;other	19.11%	6.85%	15.90%	1.99%	14.39%	3.00%	p=0.206
Odoribacter	0.46%	0.61%	0.71%	0.88%	0.29%	0.41%	p=0.319
Mucispirillum	1.87%	1.57%	1.03%	0.96%	2.76%	0.55%	p=0.234
Lactobacillus	6.34%	3.28%	11.06%	6.23%	5.80%	3.82%	p=0.295
Turicibacter	0.00%	0.00%	0.00%	0.00%	0.04%	0.07%	p=0.437
Clostridiales;other	19.58%	2.72%	18.30%	5.14%	22.91%	6.23%	p=0.450
Clostridiaceae;other	0.11%	0.07%	0.15%	0.09%	0.18%	0.09%	p=0.547
Clostridium	0.02%	0.01%	0.02%	0.02%	0.00%	0.01%	p=0.260
Lachnospiraceae;Other	6.19%	1.01%	5.86%	1.58%	10.09%	2.66%	B vs F: p=0.020
Coprococcus	0.56%	0.13%	0.98%	0.35%	0.91%	0.21%	B vs M: p=0.012 B va F: p=0.026
Ruminococcus	2.59%	0.99%	3.09%	0.48%	3.74%	1.18%	p=0.385
rc4-4	1.42%	1.81%	1.68%	2.14%	1.10%	1.35%	p=0.674
Peptostreptococcaceae;other	0.00%	0.00%	0.00%	0.01%	0.03%	0.04%	p=0.296
Ruminococcaceae;other	6.64%	2.06%	5.63%	0.85%	6.51%	1.48%	p=0.580
Oscillospira	3.95%	0.89%	4.28%	0.71%	5.02%	1.07%	p=0.258
Erysipelotrichaceae;other	0.46%	0.28%	0.45%	0.20%	0.43%	0.22%	p=0.981
Allobaculum	0.43%	0.85%	0.03%	0.04%	0.29%	0.54%	p=0.628
RF32;other	0.05%	0.08%	0.08%	0.10%	0.02%	0.03%	p=0.309
Sutterella	0.00%	0.00%	0.00%	0.01%	0.00%	0.00%	p=0.399
Bilophila	1.09%	1.27%	1.39%	1.67%	0.95%	1.23%	p=0.523
Enterobacteriaceae;other	2.77%	5.32%	0.28%	0.15%	0.18%	0.15%	p=0.454
Proteus	0.31%	0.61%	0.01%	0.01%	0.00%	0.00%	p=0.371
Akkermansia	3.34%	4.69%	2.21%	1.91%	2.61%	2.65%	p=0.808

Table S8 Relative abundance of bacteria genera between different time points in CD-1 mice fed with PL+MD

	Baseline mean	SD	Intermediate mean	SD	Final mean	SD	P value
Unassigned	3.29%	0.61%	1.80%	0.46%	2.82%	0.79%	B vs M: p=0.007 M vs F: p=0.037
Bifidobacterium	3.81%	2.02%	3.07%	1.64%	5.51%	2.31%	p=0.243
Adlercreutzia	0.49%	0.14%	0.24%	0.23%	0.34%	0.05%	B vs M: p=0.014
Bacteroides	8.85%	2.35%	34.40%	5.05%	8.74%	2.86%	B vs M: p=0.000 M vs F: p=0.000
Parabacteroides	1.44%	1.03%	0.88%	0.84%	1.18%	0.83%	p=0.560
Rikenellaceae;other	4.08%	1.85%	3.32%	1.40%	3.56%	1.71%	p=0.669
S24-7;other	20.34%	3.30%	12.33%	3.68%	14.78%	1.49%	B vs M: p=0.043
Odoribacter	0.69%	0.93%	0.04%	0.05%	0.56%	0.73%	p=0.151
Mucispirillum	1.31%	1.18%	0.83%	0.52%	0.85%	1.13%	p=0.634
Lactobacillus	2.39%	0.92%	7.22%	4.84%	8.83%	3.81%	p=0.161
Turicibacter	0.02%	0.02%	0.11%	0.21%	0.00%	0.00%	p=0.469
Clostridiales;other	22.82%	2.75%	10.90%	1.51%	21.46%	0.81%	B vs M: p=0.001 M vs F: p=0.001
Clostridiaceae;other	0.22%	0.19%	0.27%	0.24%	0.81%	1.16%	p=0.509
Clostridium	0.02%	0.02%	0.43%	0.60%	0.02%	0.02%	p=0.091
Lachnospiraceae;Other	9.06%	1.62%	3.80%	1.85%	7.99%	1.64%	B vs M: p-=0.001
Coprococcus	0.47%	0.09%	0.71%	0.08%	0.92%	0.31%	B vs F: p=0.003
Ruminococcus	2.17%	0.09%	0.97%	0.05%	2.10%	0.38%	B vs M: p=0.001 M vs F: p=0.001
rc4-4	2.09%	1.83%	0.16%	0.21%	0.61%	1.15%	B vs M: p=0.024
Peptostreptococcaceae;other	0.33%	0.66%	0.14%	0.16%	0.56%	0.53%	p=0.584
Ruminococcaceae;other	7.35%	2.30%	2.96%	0.79%	6.00%	1.48%	B vs M: p=0.034
Oscillospira	4.96%	1.10%	2.51%	0.64%	6.13%	0.84%	B vs M: p=0.032 M vs F: p=0.005
Erysipelotrichaceae;other	1.01%	0.55%	2.11%	0.82%	0.71%	0.71%	p=0.085
Allobaculum	0.07%	0.14%	1.52%	2.29%	1.07%	1.23%	p=0.503
RF32;other	0.14%	0.14%	0.48%	0.58%	0.05%	0.09%	B vs M:p=0.029 M vs F: p=0.011
Sutterella	0.00%	0.00%	0.29%	0.52%	0.00%	0.01%	p=0.381
Bilophila	0.54%	0.94%	0.79%	0.91%	0.99%	1.18%	p=562
Enterobacteriaceae;other	0.09%	0.06%	0.49%	0.22%	0.06%	0.04%	B vs M: p=0.023 M vs F: p=0.017
Proteus	0.00%	0.00%	0.00%	0.00%	0.01%	0.01%	p=0.530
Akkermansia	1.96%	2.02%	7.23%	5.22%	3.37%	3.73%	p=0.276

Table S9 Relative abundance of bacteria genera between different time points in CD-1 mice fed with P+MD

	Baseline mean	SD	Intermediate mean	SD	Final mean	SD	P value
Unassigned	2.84%	0.41%	2.34%	0.80%	1.81%	0.59%	p=0.094
Bifidobacterium	5.38%	3.20%	4.72%	3.27%	4.33%	3.69%	p=0.911
Adlercreutzia	0.48%	0.19%	0.23%	0.03%	0.26%	0.07%	B vs M: p=0.035
Bacteroides	8.63%	1.68%	16.66%	12.35%	15.97%	7.87%	p=0.280
Parabacteroides	1.78%	1.40%	0.89%	0.75%	1.10%	0.99%	p=0.395
Rikenellaceae;other	4.48%	1.80%	3.31%	0.97%	4.52%	1.84%	p=0.435
S24-7;other	18.69%	2.98%	14.37%	3.29%	19.84%	3.03%	p=0.063
Odoribacter	0.47%	0.54%	0.18%	0.21%	0.35%	0.48%	p=0.202
Mucispirillum	1.44%	1.31%	1.40%	0.47%	1.64%	1.17%	p=0.934
Lactobacillus	4.79%	2.42%	4.66%	3.47%	3.38%	2.51%	p=0.663
Turicibacter	0.19%	0.38%	0.81%	1.07%	0.25%	0.30%	p=0.184
Clostridiales;other	24.36%	2.07%	22.38%	8.12%	25.10%	4.27%	p=0.823
Clostridiaceae;other	0.68%	1.20%	0.11%	0.11%	0.08%	0.06%	p=0.443
Clostridium	0.02%	0.03%	0.10%	0.19%	0.00%	0.00%	p=0.450
Lachnospiraceae;Other	6.15%	0.66%	6.26%	1.47%	6.81%	2.86%	p=0.820
Coprococcus	0.63%	0.23%	0.67%	0.26%	0.84%	0.47%	p=0.070
Ruminococcus	2.32%	0.58%	1.63%	0.72%	1.14%	0.26%	B vs F: p=0.012
rc4-4	1.94%	1.71%	1.04%	1.21%	1.62%	1.92%	B vs M: p=0.029
Peptostreptococcaceae;other	0.01%	0.02%	0.07%	0.07%	0.01%	0.02%	p=0.284
Ruminococcaceae;other	5.70%	0.80%	5.07%	3.31%	3.04%	0.85%	p=0.169
Oscillospira	5.09%	1.65%	3.70%	1.86%	3.13%	1.24%	p=0.283
Erysipelotrichaceae;other	0.41%	0.15%	0.96%	0.71%	0.42%	0.35%	p=0.332
Allobaculum	0.19%	0.38%	1.66%	2.08%	0.80%	1.01%	p=0.396
RF32;other	0.11%	0.14%	0.12%	0.15%	0.46%	0.79%	p=0.460
Sutterella	0.00%	0.00%	0.17%	0.29%	0.16%	0.30%	p=0.560
Bilophila	0.93%	1.12%	0.82%	0.94%	1.20%	1.38%	p=0.108
Enterobacteriaceae;other	1.72%	3.06%	0.22%	0.32%	0.73%	1.36%	p=0.581
Proteus	0.00%	0.00%	0.00%	0.00%	0.00%	0.00%	p=0.732
Akkermansia	0.59%	0.82%	5.46%	3.49%	1.01%	0.74%	B vs M: p=0.002 M vs F: p=0.004

Table S10 Relative abundance of bacteria genera between different time points in CD-1 mice fed with PL+P+MD

	Baseline mean	SD	Intermediate mean	SD	Final mean	SD	P value
Unassigned	3.13%	1.05%	1.81%	0.37%	1.77%	0.78%	p=0.036
Bifidobacterium	4.98%	2.44%	6.17%	4.87%	1.42%	0.86%	p=0.243
Adlercreutzia	0.40%	0.08%	0.29%	0.10%	0.21%	0.12%	p=0.159
Bacteroides	11.69%	2.51%	12.56%	3.92%	26.25%	11.49%	p=0.085
Parabacteroides	3.44%	3.03%	0.79%	0.41%	0.55%	0.45%	B vs M: p=0.000 B vs F: p=0.000
Rikenellaceae;other	4.94%	0.71%	3.24%	1.67%	3.23%	1.08%	p=0.129
S24-7;other	20.08%	0.88%	19.75%	4.93%	17.59%	7.44%	p=0.800
Odoribacter	0.73%	0.85%	0.61%	0.74%	0.45%	0.84%	p=0.747
Mucispirillum	0.67%	0.73%	1.99%	1.43%	1.86%	3.06%	p=0.629
Lactobacillus	3.05%	2.21%	2.40%	1.53%	2.90%	3.09%	p=0.933
Turicibacter	0.00%	0.01%	0.00%	0.00%	0.00%	0.00%	p=0.468
Clostridiales;other	18.34%	2.82%	20.45%	2.49%	13.75%	4.27%	p=0.078
Clostridiaceae;other	0.22%	0.09%	0.06%	0.07%	0.05%	0.05%	p=0.43
Clostridium	0.01%	0.00%	0.00%	0.00%	0.01%	0.02%	p=0.577
Lachnospiraceae;Other	6.35%	0.71%	6.18%	1.48%	5.73%	1.97%	p=0.862
Coprococcus	0.48%	0.19%	0.90%	0.33%	0.76%	0.32%	p=0.168
Ruminococcus	3.20%	2.63%	2.23%	1.32%	1.36%	0.68%	p=0.407
rc4-4	2.82%	2.91%	0.77%	0.94%	0.48%	0.77%	B vs M: p=0.035 B vs F: p=0.020
Peptostreptococcaceae;other	0.35%	0.69%	0.00%	0.01%	0.15%	0.24%	p=0.542
Ruminococcaceae;other	6.44%	2.20%	5.18%	2.10%	2.91%	1.07%	p=0.073
Oscillospira	3.56%	0.40%	4.70%	1.52%	2.44%	0.86%	M vs F: p=0.006
Erysipelotrichaceae;other	1.14%	0.87%	1.97%	1.55%	2.49%	1.20%	p=0.335
Allobaculum	0.03%	0.06%	0.74%	0.66%	0.85%	0.54%	p=0.044
RF32;other	0.16%	0.20%	0.13%	0.16%	0.19%	0.22%	p=0.571
Sutterella	0.02%	0.03%	0.02%	0.02%	0.33%	0.27%	p=0.102
Bilophila	1.46%	1.75%	1.12%	1.40%	1.53%	1.80%	p=0.577
Enterobacteriaceae;other	0.54%	0.74%	0.03%	0.02%	0.38%	0.47%	p=0.394
Proteus	0.26%	0.52%	0.00%	0.00%	0.00%	0.00%	p=0.424
Akkermansia	1.50%	1.90%	5.88%	5.61%	10.38%	5.41%	p=0.140

Table S11. Relative abundance of bacteria phylum between female and male mice

	Female mean	SD	Male mean	SD	p value
Unassigned;Other	0.92%	0.37%	0.55%	0.29%	P=0.000
Actinobacteria	4.11%	3.67%	5.32%	4.15%	p=0.328
Bacteroidetes	38.37%	7.57%	37.07%	10.60%	P=0.463
Deferribacteres	1.61%	1.52%	1.33%	1.09%	P=0.489
Firmicutes	47.52%	8.17%	51.52%	11.68%	P=0.074
Proteobacteria	2.52%	1.09%	1.59%	2.91%	P=0.172
Verrucomicrobia	4.96%	4.18%	2.63%	4.02%	P=0.037

Table S12. Relative abundance of bacterial genera between female and male mice

	Female mean	SD	Male mean	SD	p value
Unassigned	2.38%	0.76%	2.76%	0.99%	P=0.000
Bifidobacterium	3.71%	3.61%	4.79%	3.97%	P=0.368
Adlercreutzia	0.37%	0.14%	0.39%	0.26%	P=0.615
Bacteroides	13.30%	8.21%	15.35%	10.87%	P=0.252
Parabacteroides	2.47%	1.78%	0.52%	0.29%	P=0.000
Rikenellaceae;other	4.98%	1.81%	3.25%	1.27%	P=0.001
S24-7;other	17.61%	5.07%	16.92%	3.79%	P=0.555
Odoribacter	0.00%	0.00%	0.92%	0.58%	P=0.000
Mucispirillum	1.61%	1.52%	1.33%	1.09%	P=0.489
Lactobacillus	6.13%	4.16%	4.34%	3.76%	P=0.100
Turicibacter	0.02%	0.09%	0.21%	0.51%	P=0.011
Clostridiales;other	19.36%	5.06%	20.70%	5.86%	P=0.312
Clostridiaceae;other	0.13%	0.14%	0.36%	0.67%	P=0.126
Clostridium	0.01%	0.01%	0.10%	0.27%	P=0.031
Lachnospiraceae;Other	6.47%	1.66%	6.95%	2.67%	P=0.257
Coprococcus	0.74%	0.22%	0.73%	0.36%	P=0.989
Ruminococcus	2.46%	1.47%	1.96%	0.88%	P=0.119
rc4-4	0.16%	0.28%	2.46%	1.58%	P=0.000
Peptostreptococcaceae;other	0.15%	0.35%	0.12%	0.33%	P=0.776
Ruminococcaceae;other	5.12%	2.12%	5.45%	2.22%	P=0.504
Oscillospira	3.95%	1.44%	4.29%	1.52%	P=0.289
Erysipelotrichaceae;other	1.02%	0.81%	1.07%	1.14%	P=0.804
Allobaculum	0.40%	1.03%	0.88%	1.12%	P=0.159
RF32;other	0.01%	0.02%	0.32%	0.38%	P=0.000
Sutterella	0.04%	0.13%	0.13%	0.28%	P=0.178
Bilophila	2.13%	0.77%	0.01%	0.01%	P=0.000
Enterobacteriaceae;other	0.32%	0.57%	0.93%	2.45%	P=0.259
Proteus	0.00%	0.01%	0.10%	0.32%	P=0.176
Akkermansia	4.96%	4.18%	2.63%	4.02%	P=0.037
