# Supplementary material for: Food-grade cationic antimicrobial ε-polylysine transiently alters the gut microbial community and predicted metagenome function in CD-1 mice
Source: NPJ Sci Food. 2017 Dec 18;1:8. doi: 10.1038/s41538-017-0006-0 (PMC6550245; doi:10.1038/s41538-017-0006-0)
Supplement: Supplementary file 2 — Supplemental figures [file 41538_2017_6_MOESM2_ESM.pdf]

SM Figure

Fig. S1

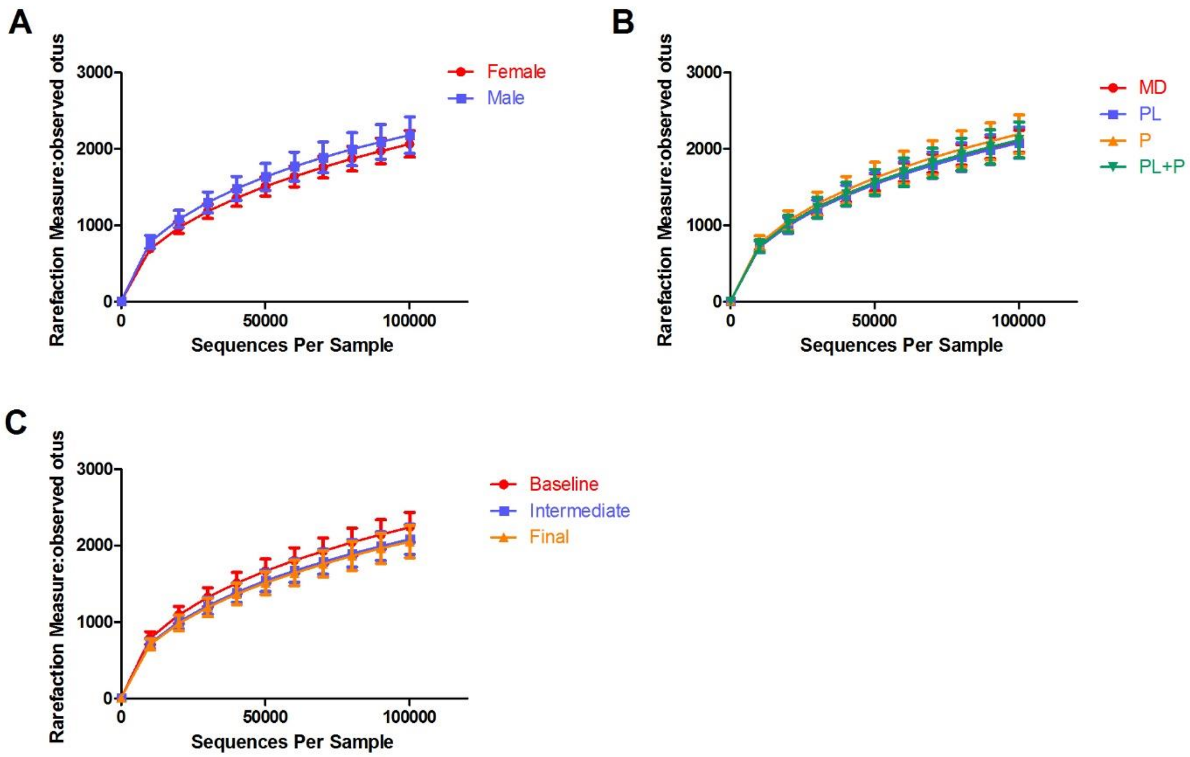

Rarefication curves for observed OTUs by sex (a), treatments (b), and time points (c).

Fig. S2

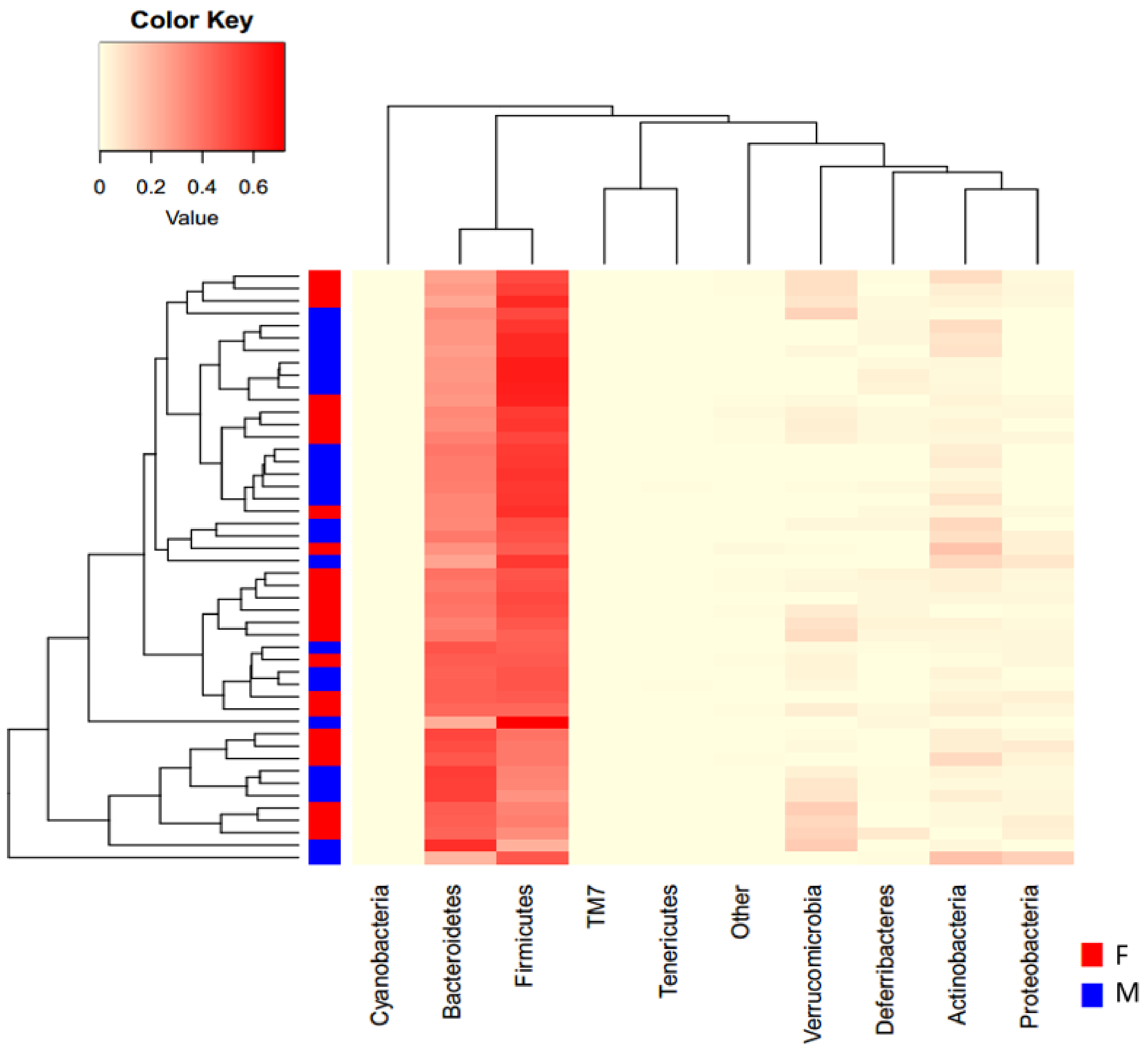

Hierarchical clustering of samples using taxonomic profiles at the phylum level by sex. F, female M, male

Fig. S3

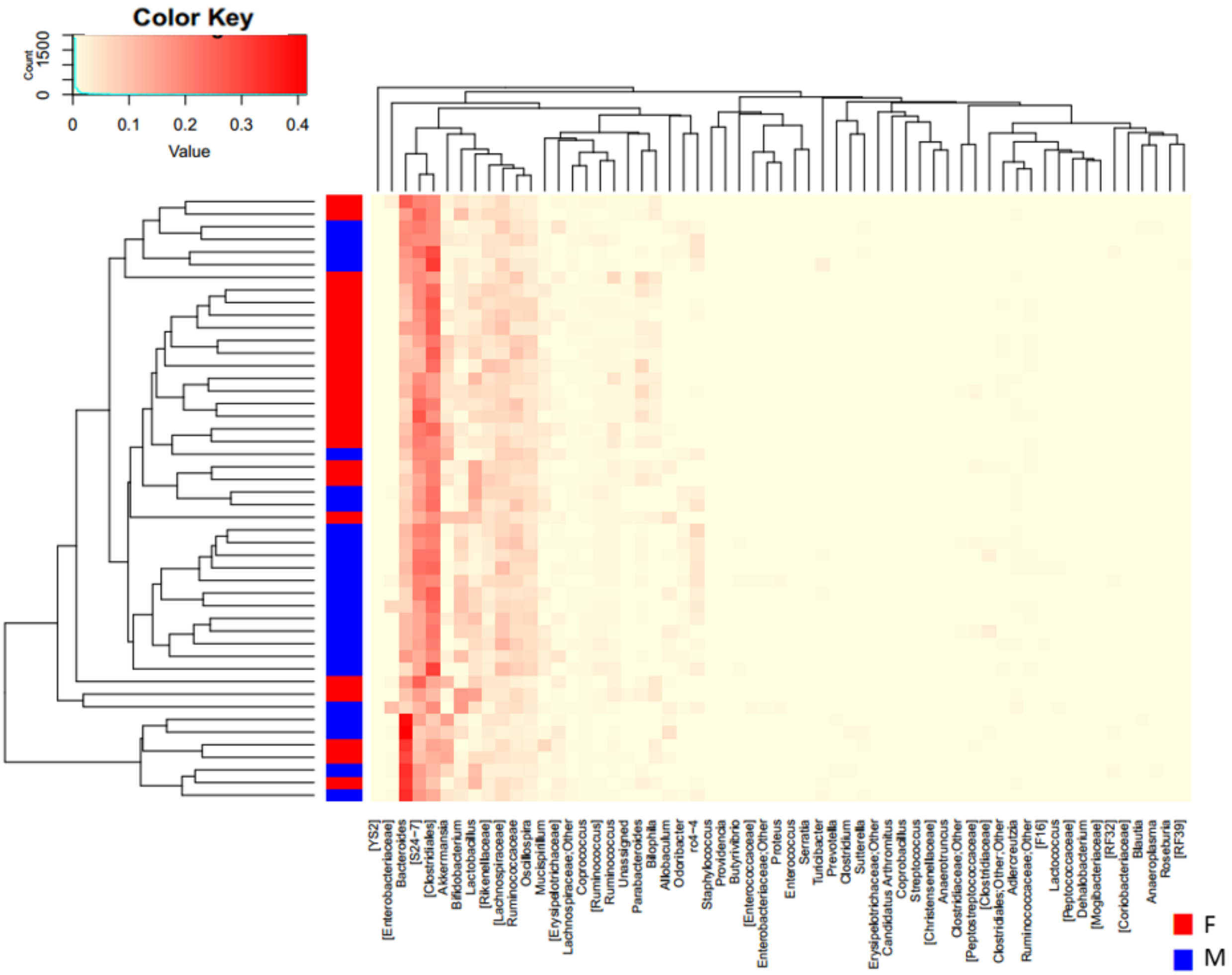

Hierarchical clustering of samples using taxonomic profiles at the genus level by sex. F, female M, male

Fig. S4

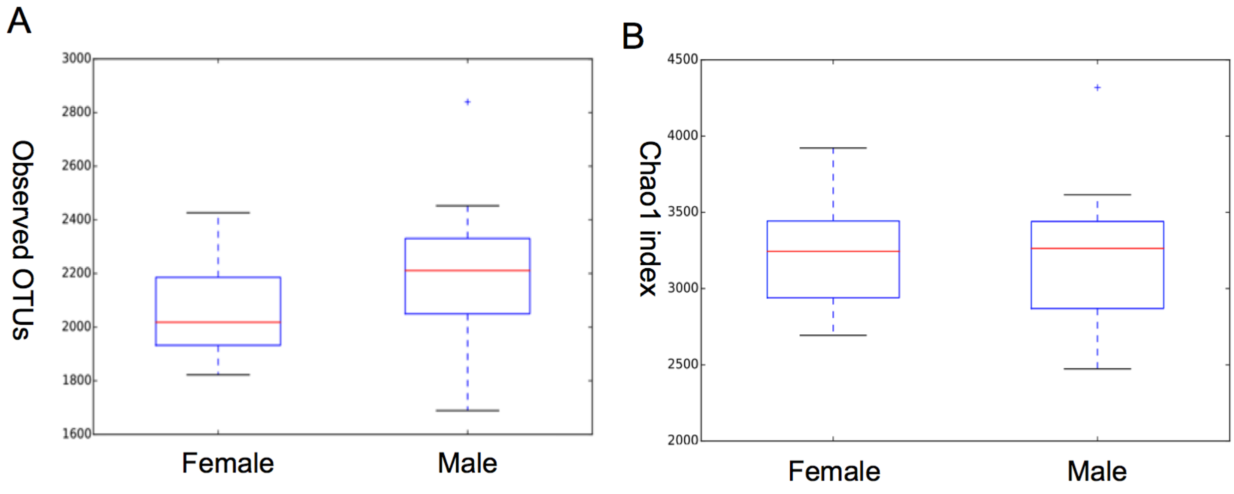

Alpha diversity by sex (a) observed OTUs (b) Chao1 index.

Fig. S5

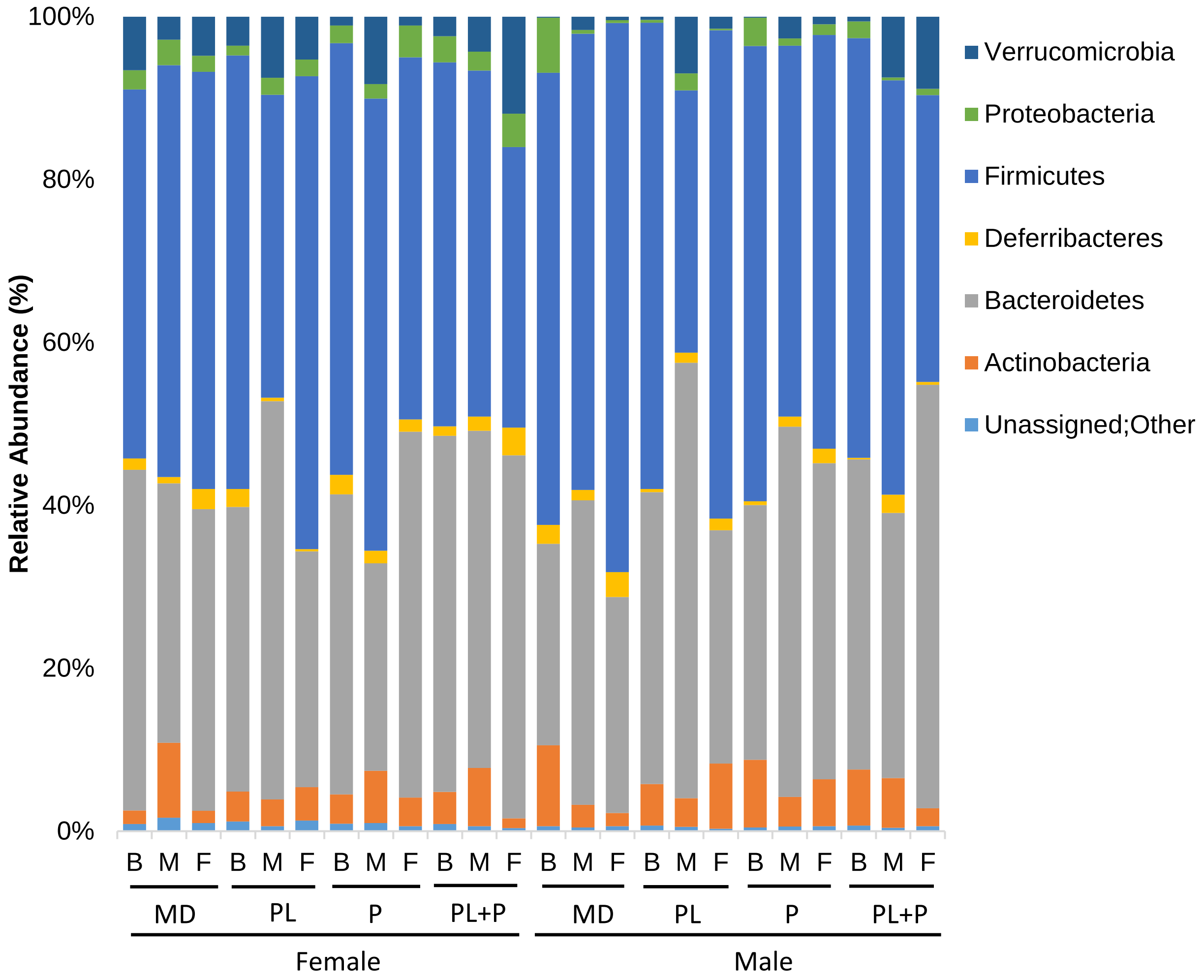

Relative abundance of bacterial phylum in response to biopolymer treatments by sex. B, baseline M, intermediate F, final MD, maltodextrin PL, ε-polylysine P, pectin PL+P, ε-polylysine-pectin complexes

Fig. S6

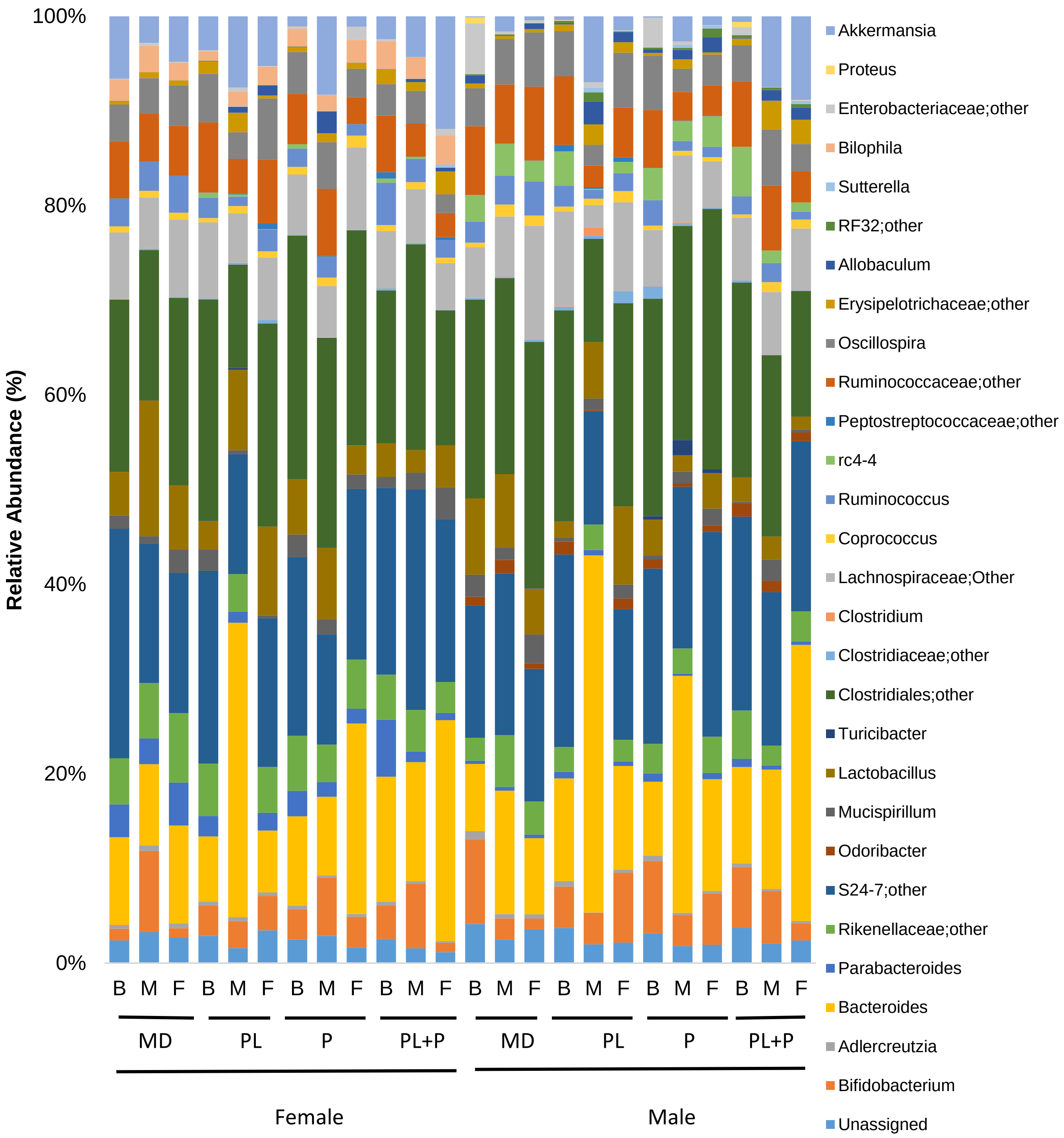

Relative abundance of bacterial genus in response to biopolymer treatments by sex. B, baseline M, intermediate F, final MD, maltodextrin PL, ε-polylysine P, pectin PL+P, ε-polylysine-pectin complexes
